# Interleukin-17 family in health and immune diseases: From origin to clinical implications

**DOI:** 10.4103/NRR.NRR-D-25-00026

**Published:** 2025-06-19

**Authors:** Guozhen Deng, Mengdi Guo, Jiahui Fan, Weiyan Wang, Mei-Ling Jiang, Cun-Jin Zhang

**Affiliations:** 1 Department of Neurology, Sichuan Provincial People’s Hospital, University of Electronic Science and Technology of China, Chengdu, Sichuan Province, China; 2 Department of Science and Technology, Sichuan Provincial People’s Hospital, University of Electronic Science and Technology of China, Chengdu, Sichuan Province, China

**Keywords:** antibody therapy, autoimmune disease, cellular source, clinical applications, interleukin-17, interleukin-17 receptor, inflammatory diseases, physiological responses, signaling pathway, therapeutic strategy

## Abstract

The interleukin-17 family is the key group of cytokines and displays a broad spectrum of biological functions, including regulating the inflammatory cascade in various autoimmune and inflammatory diseases, such as multiple sclerosis, neuromyelitis optica spectrum disorder, myasthenia gravis, Guillain–Barre syndrome, acute disseminated encephalomyelitis, diabetes, inflammatory skin diseases, joint inflammation, and cancer. Although the function of the interleukin-17 family has attracted increasing research attention over many years, the expression, function, and regulation mechanisms of different interleukin-17 members are complicated and still only partially understood. Currently, the interleukin-17A pathway is considered a critical therapeutic target for numerous immune and chronic inflammatory diseases, with several monoclonal antibodies against interleukin-17A having been successfully used in clinical practice. Whether other interleukin-17 members have the potential to be targeted in other diseases is still debated. This review first summarizes the recent advancements in understanding the physicochemical properties, physiological functions, cellular origins, and downstream signaling pathways of different members and corresponding receptors of the interleukin-17 family. Subsequently, the function of interleukin-17 in various immune diseases is discussed, and the important role of interleukin-17 in the pathological process of immune diseases is demonstrated from multiple perspectives. Then, the current status of targeted interleukin-17 therapy is summarized, and the effectiveness and safety of targeted interleukin-17 therapy are analyzed. Finally, the clinical application prospects of targeting the interleukin-17 pathway are discussed.

## Introduction

Interleukin (IL)-17A was first identified and reported in 1993 through the isolation of cytokines from a T lymphocyte fusion cell (Rouvier et al., 1993), while its IL-17A receptor (IL-17RA) was initially identified in 1995 (Yao et al., 1995a). IL-17A and IL-17RA exhibit unique molecular structures with sequences distinct from all other known mammalian cytokines (Liu, 2019). Through subsequent expression sequence tagging and large-scale genome sequencing of several vertebrates, other members of the IL-17 family were consecutively identified. In total, six independent and distinct entities of the IL-17 cytokine group have been identified, ranging from IL-17A to IL-17F. The IL-17 cytokine group exhibits a high degree of structural similarity, typically including a conserved domain known as the “cysteine knot fold” (Gunimaladevi et al., 2006). This structural domain exhibits a high degree of homology across different members. The peptide sequences within the domain of IL-17 family members exhibit significant homology, with approximately 20%–50% sequence similarity between different members, while maintaining sufficient diversity to confer distinct biological functions and specificities (Gu et al., 2013). This structural conservation enables these cytokines to interact with their receptors through similar mechanisms and exert their biological effects. Additionally, IL-17 family members generally exist in the form of dimers and multimers, a structural characteristic that plays a critical role in their functional activity. IL-17A and IL-17F, for instance, function as dimers to bind receptors and activate signaling pathways, whereas IL-17B may form other types of aggregates (Bie et al., 2017). The formation of dimers or multimers enhances their receptor-binding affinity and signaling efficiency. The IL-17 receptor (IL-17R) family currently comprises five members, from IL-17RA to IL-17E receptors (IL-17RE) (Liu, 2019). Each receptor contains a common structural region, “SEFIR” (SEF/interleukin-17 receptors), which shares similarities with the Toll/interleukin-1 receptor (TIR) domain found in Toll-like receptors (TLRs) and IL-1 receptors (Yao et al., 1995b). The SEFIR domain can recruit adaptor proteins, such as nuclear factor κB (NF-κB) activator 1 (Act1) and tumor necrosis factor receptor-associated factor 6 (TRAF6), which are instrumental in activating downstream signaling pathways. Additionally, through the action of the SEFIR domain, IL-17Rs can trigger NF-κB and mitogen-activated protein kinase (MAPK) signaling, leading to the upregulation of inflammatory mediators and intensifying the immune response. The IL-17 family is acknowledged as the pioneer of an evolutionarily conserved class of signaling molecules.

IL-17 has been tightly linked to inflammation and autoimmune diseases, with its initial expression observed in T helper 17 (Th17) cells (Singh Gautam and Kumar Singh, 2023). The lineage commitment of Th17 cells is governed by a two-phase regulatory program: (1) Differentiation initiation driven by transforming growth factor-β (TGF-β) synergizing with IL-6 or IL-21 to induce RORγT (retinoic acid-related orphan receptor gamma t) expression through signal transducer and activator of transcription 3 (STAT3), which establishes the transcriptional foundation for Th17 identity (Whitley et al., 2018); and (2) Phenotype stabilization mediated by IL-23 signaling, which reinforces RORγt activity via STAT3-dependent epigenetic remodeling while upregulating IL-23 receptor expression, creating a self-sustaining loop critical for pathogenic functionality. This bifurcated regulation is evolutionarily conserved to prevent aberrant Th17 activation, as evidenced by RORγt-deficient mice demonstrating complete ablation of Th17 development despite intact IL-23 signaling (Ivanov et al., 2006). The “IL-23/IL-17 axis” is recognized as a significant contributor to autoimmune diseases (Stein et al., 2021). Beyond Th17 cells, IL-17 is also secreted by clusters of differentiation (CD)8^+^ (Tc17) cells, γδ T cells, natural killer T cells, group 3 innate lymphoid cells (ILC3), along with “natural” Th17 cells, subsets of innate lymphocytes (Feng et al., 2023). IL-17 mediates its effects via directly binding to IL-17Rs, which then induce target gene transcription via the adaptor protein NF-κB activator 1 (Act1) and its downstream molecules, including TRAF protein activation, NF-κB, CCAAT/enhancer binding protein beta (C/EBPβ), CCAAT/enhancer binding protein delta (C/EBPδ), and MAPK signaling cascades. This classic signaling is critical for IL-17-induced tissue inflammation, autoimmune responses, and adaptive immune reactions. Mounting evidence supports the crucial contribution of IL-17 to the development of multiple sclerosis (MS), rheumatoid arthritis (RA), and other autoimmune diseases. Monoclonal antibodies designed to target IL-17A, IL-17F, IL-17RA, and IL-23 have been authorized for clinical use in treating IL-17-associated autoimmune disorders, such as psoriasis (Winthrop et al., 2024).

**Figure 1:**
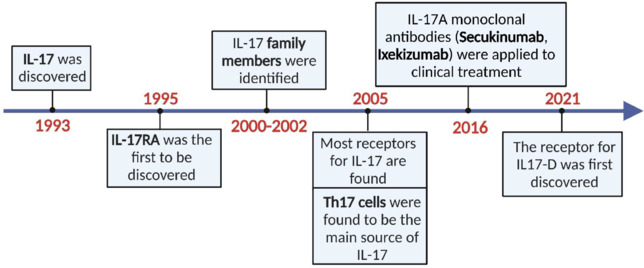
Timeline of the progress of IL-17 research. The milestone map delineates a chronological series of pivotal events, including the elucidation of the origin of IL-17, discoveries of other IL-17 family members and their cognate receptors, identification of IL-17-producing cellular sources, clinical applications of IL-17A-targeting monoclonal antibody inhibitors, and recent breakthroughs in receptor identification for IL-17D. Created with BioRender.com. IL-17: Interleukin 17; IL-17A: interleukin 17A; IL-17D: interleukin 17D; IL-17RA: interleukin 17A receptor.

IL-17 acts as an essential cytokine, a key pathological mediator, and a therapeutic target in numerous autoimmune diseases. We summarized the function of the IL-17 family in initiating autoimmune inflammation within pathological contexts and its contribution to the development and progression of various inflammatory diseases. The most recent findings related to the IL-17 signaling network offer an in-depth examination of its crosstalk with other signaling molecules and present a perspective on targeting IL-17 as a potential strategy for treating several inflammatory diseases (**Figure 1**).

## Search Strategy

An extensive literature review was performed across PubMed, Scopus, and Web of Science using key terms such as “IL-17,” “Th17,” “autoimmune diseases,” “neuroinflammation,” and “monoclonal antibodies.” Studies published between 1993 and 2025 focused on the molecular processes of IL-17 cytokines, their involvement in inflammation and tissue repair, and the potential for therapeutic strategies aimed at modulating the IL-17/IL-17R pathway. Clinical trials, animal models, and review articles were also included to provide a comprehensive understanding of the therapeutic implications of IL-17.

## Interleukin-17 Family Members and Receptors

IL-17A was first identified as cytotoxic T lymphocyte antigen-8 (CTLA-8) (Shin et al., 1998) and is considered the initial cytokine discovered in the IL-17 cytokine groups. Its complementary DNA encodes 150 polypeptides, showing 57% similarity with the open reading frame 13 protein from Semiri herpes virus but no similarity to other cytokine families (Rouvier et al., 1993). IL-17A is a dimeric protein, either as a homodimer (IL-17A/IL-17A) or a heterodimer (IL-17A/IL-17F), with each monomer weighing approximately 15–20 kDa (Jovanovic et al., 1998; Johansen et al., 2009). High-resolution crystallographic studies have classified IL-17A within the cysteine-knot fold structural family (Weaver et al., 2007), which is characterized by a distinctive bouquet-like β-fold architecture. This structural pattern can also be observed for other cytokines, such as nerve growth factor and TGF-β, although the β-fold in IL-17A is notably more compact (Novatchkova et al., 2003; Chen et al., 2019a). Every unit of IL-17A consists of a pair of primary β-sheet layers, the structural integrity of which is reinforced by multiple conserved cysteine residues that form intramolecular disulfide bonds (Huang et al., 2023). IL-17RA and IL-17RC serve as the primary receptors for IL-17A. As type I membrane proteins, they are expressed in a variety of cellular populations, such as epithelial cells, fibroblasts, and endothelial and immune cells (Hu et al., 2010; Robert and Miossec, 2024). IL-17RA acts as the main binding subunit for IL-17A and is widely expressed, facilitating critical signaling for multiple IL-17 family members. It features an extended intracellular domain that plays a key role in recruiting downstream signaling molecules. In contrast, IL-17RC exhibits more tissue-specific expression and typically partners with IL-17RA to create the receptor complex essential for signal transduction (Kuestner et al., 2007). Furthermore, the extracellular domain of IL-17RC aids in IL-17A binding, providing additional support (Toy et al., 2006; You et al., 2006). IL-17A signaling is triggered as IL-17R interacts with Act1 via its intracellular SEFIR domain. Act1 is a critical cytoplasmic adaptor that initiates every identified IL-17-dependent signaling cascade. It features a SEFIR domain, which enables its interaction with stress-activated protein kinase, a key component in the signaling process (Sønder et al., 2011; Zhang et al., 2014). Beyond the SEFIR domain, IL-17RA has been found to possess a nonconserved region referred to by the name “SEFIR extension” (SEFEX), which is crucial for ensuring the correct execution of its signaling role in IL-17RA signaling (Song et al., 2023). Moreover, the intracellular region of IL-17RA encompasses a separate segment independent of the SEFIR and SEFEX domains, which are crucial for initiating C/EBPβ; this segment is known as the C/EBPβ activation domain (CBAD) (Dzielski and Kotanchek, 1998; Song and Qian, 2013). Together, these domains ensure the effectiveness and specificity of IL-17 pathway signaling.

Between 2000 and 2002, an additional five cytokines were identified as part of the IL-17 family: IL-17B (Li et al., 2000), IL-17C (Li et al., 2000), IL-17D (Starnes et al., 2002), IL-17E (Lee et al., 2001), and IL-17F (Hymowitz et al., 2001). IL-17B was initially reported to have elevated expression levels during intestinal inflammation (Zhang et al., 2019). Li et al. (2000) first cloned and expressed human IL-17B, which showed 88% similarity to its mouse counterpart, with just 29% similarity to human IL-17A. IL-17B, located on chromosome 5q32-34, is predominantly found in human tissues such as the pancreas, small intestine, and stomach. IL-17RB is the primary receptor for IL-17B and is capable of mediating the activation of downstream signals and regulating inflammatory and immune responses. After peripheral nerve injury, Huang et al. (2024) reported the activation of IL-17B signaling through its receptor, IL-17RB, in Schwann cells. This process stimulates the production of signaling molecules that recruit macrophages, aiding in myelin debris clearance and promoting axon regeneration. A mouse model indicated that IL-17B suppresses B-cell activation and differentiation by downregulating fatty acid synthase-mediated lipid metabolism, thereby suppressing B-cell activation and differentiation. This greatly reduces symptoms in mice susceptible to lupus. Furthermore, compared with those in the control group, B cells from systemic lupus erythematosus (SLE) patients presented increased levels of fatty acid synthase and decreased levels of IL-17RB (Xiao et al., 2024).

Moreover, IL-17C was initially discovered by searching for proteins related to IL-17A, and its gene is located on chromosome 16q24 and shares 27% homology with IL-17A (Li et al., 2000). In contrast to other IL-17 family cytokines, IL-17C is a proinflammatory protein produced by epithelial cells instead of hematopoietic cells, but the specific transcriptional regulatory mechanisms involved remain unclear (Ramirez-Carrozzi et al., 2011). A considerable amount of research shows that IL-17C is highly upregulated during the initial phases of the disease (Song et al., 2011; Krohn et al., 2018). Notably, IL-17C is significantly elevated in the skin lesions of psoriasis patients and atopic dermatitis (AD) patients. In psoriasis skin lesions, the level of IL-17C is approximately two orders of magnitude greater than that of IL-17A (Vandeghinste et al., 2018). Research has demonstrated that IL-17C specifically binds to IL-17RE, as demonstrated by the transfection of 293T cells with the IL-17RA-RE receptor complex (Chang et al., 2011). Additionally, Song et al. (2011) reported that IL-17C can also bind to the heterodimer formed by IL-17RA and IL-17RE. Jiang et al. (2024) demonstrated that IL-17C, either alone or in combination with tumor necrosis factor α (TNF-α) and IL-17A, reduces the expression of transcription factor 4 in an IL-17RA/RE-dependent manner, thereby promoting skin inflammation.

IL-17D is the most underresearched member of the IL-17F family, with its gene first identified in 2002 and then mapped to chromosome 13p11 (Starnes et al., 2002). As the largest member of the IL-17 family, IL-17D is a glycoprotein composed of 202 amino acids. Its monomeric form has a predicted molecular weight of 26.3 kDa. Like other family members, IL-17D likely forms homodimers through intermolecular disulfide bonds mediated by four conserved cysteine residues. Functionally, IL-17D stimulates endothelial cells to produce cytokines such as IL-6, IL-8, and granulocyte‒macrophage colony‒stimulating factor (GM-CSF) (Starnes et al., 2002). It also induces the expression of hematopoietic growth factors and chemokines, thereby increasing local leukocyte infiltration and proliferation, which underscores its critical role in orchestrating localized immune responses. Notably, the immunomodulatory functions of IL-17D appear to depend on its activation of canonical signaling pathways. In lung cancer cells overexpressing IL-17D, upregulation of chemokine (C–C motif) ligand (CCL) 3/4 and colony stimulating factor 1 has been observed. These genes are associated with macrophage recruitment and polarization, accompanied by robust phosphorylation of p38 MAPK, indicating that IL-17D activates the p38 MAPK signaling pathway to promote tumor-associated macrophage infiltration (Lin et al., 2022b). Similarly, in inflammatory skin diseases, IL-17D regulates keratinocyte-mediated inflammatory responses through the CD93‒p38 MAPK‒protein kinase B (AKT)-Smad2/3 signaling axis (Ni et al., 2022). IL-17D does not bind to any known IL-17R family members, including both homodimeric and heterodimeric forms. However, IL-17D was found to bind to RAW264.7 cells, suggesting the presence of a yet-to-be-identified receptor on these cells. The identification of the IL-17D receptor was conducted via immunoprecipitation-mass spectrometry in RAW264.7 cells. From the mass spectrometry data, over 200 proteins were identified, but only 293T cells overexpressing CD93 were capable of binding to IL-17D. Moreover, CD93 was confirmed to be an IL-17D receptor, showing an affinity comparable to that of the interaction of IL-17 with its receptor, IL-17RA (Huang et al., 2021). CD93 (cluster of differentiation 93), also known as C1qR1 or C1qRP, is a transmembrane glycoprotein composed of 652 amino acids. It is considered a cell surface receptor for the complement component C1q. However, paradoxically, a report suggested that CD93 does not bind to C1q. CD93 is widely expressed on the membranes of various cell types, including endothelial cells, hematopoietic stem cells, and platelets, as well as certain immune cells, such as B cells, neutrophils, and natural killer cells (McGreal et al., 2002; Borah et al., 2019). Starting from the N-terminus, CD93 features a conserved C-type lectin-like domain characterized by eight highly conserved cysteine residues, followed by a sushi-like domain, five epidermal growth factor-like domains, and a mucin-like domain that likely contains multiple glycosylation sites. Near the C-terminus, there is a transmembrane domain, along with an intracellular cytoplasmic tail (Khan et al., 2019). Among these domains, the region between the C-type lectin-like domain and epidermal growth factor-like domains is essential for IL-17D binding (Huang et al., 2021).

IL-17E is a distinct member of the IL-17 family. It was first discovered in 2001 and is located on chromosome 14q11 (Fort et al., 2001). Moreover, IL-17E, a member of the IL-17 cytokine group, shares the lowest degree of homology with IL-17A, at only 16% (Lee et al., 2001). IL-17RB must interact with IL-17RA to interact with IL-17E, triggering molecular cascades that elicit responses in target cells (Borowczyk et al., 2021). Owing to its unique role in regulating type 2 immune responses and its ability to promote the activation of Th2 cells and the secretion of associated cytokines, IL-17E was renamed IL-25 to better differentiate its mechanism of action from that of other proinflammatory cytokines within the family (Huang et al., 2015; von Moltke et al., 2016). Although it is functionally distinct from other IL-17 members, IL-25 maintains homology in terms of molecular structure and evolutionary lineage. Consequently, IL-25 is still considered a member of the IL-17 family, albeit with a particularly specialized role in immune regulation (Chang and Dong, 2011). Recently, Wilson et al. (2022) studied the structures of binary and ternary complexes involving IL-25 and its receptors and revealed that IL-25 specifically binds to IL-17RB. This interaction facilitates the establishment of the ‘tip-to-tip’ connection between IL-17RB and IL-17RA in an allosteric manner, revealing the key organizing structural principles of the IL-17R group. Indeed, IL-17E is known to strongly drive type 2 immunity, bolster epithelial defenses, and maintain immune balance (Fort et al., 2001). Blocking IL-25/IL-17RA decreases the infiltration of lymphocytes into organs, thereby preventing the development of immune-related side effects, such as pneumonitis and hepatitis (Hu et al., 2024b). In diabetic mouse models and human umbilical vein endothelial cells, the IL-25-activated IL-17RB pathway restored the reduction in Wnt/β-catenin signaling and triggered the phosphorylation of AKT and extracellular signal-related kinase (ERK) 1/2 in a high-glucose environment, ultimately promoting diabetic wound healing (Zhang et al., 2022a).

**Figure 2:**
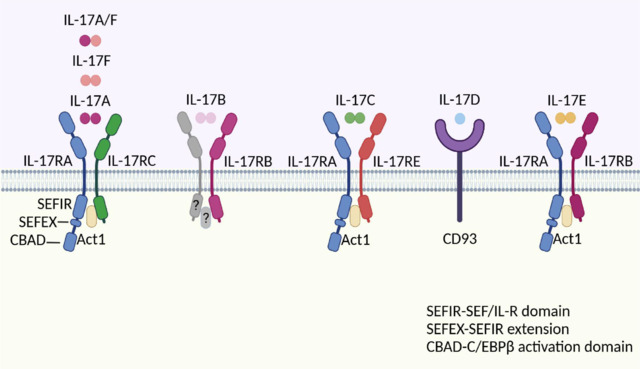
Schematic overview of the IL-17 family members and their respective receptor complexes. IL-17 cytokines, which are characterized by a cysteine-knot structure, bind to specific IL-17R receptors, activating immune signaling via the SEFIR, SEFEX, and CBAD domains. IL-17A/F signals through IL-17RA/RC, whereas IL-17C and IL-17E use IL-17RA/RE and IL-17RA/RB, respectively. CD93 was identified as the receptor for IL-17D. Created with BioRender.com. Act1: Nuclear factor κB activator 1; CBAD: CCAAT/enhancer binding protein beta activation domain; CD93: cluster of differentiation 93; IL-17: interleukin 17; IL-17A: interleukin-17A; IL-17B: interleukin-17B; IL-17C: interleukin-17C; IL-17D: interleukin-17D; IL-17E: interleukin-17E; IL-17F: interleukin-17F; IL-17R: interleukin-17 receptor; IL-17RA: interleukin-17A receptor; IL-17RB: interleukin-17B receptor; IL-17RC: interleukin-17C receptor; IL-17RE: interleukin-17E receptor; SEFIR: similar expression to the fibroblast growth factor-IL-1 receptor; SEFEX: SEFIR extension.

IL-17F is approximately 50% similar to IL-17A and can form either a homodimer (IL-17F/IL-17F) or a heterodimer with IL-17A (IL-17A/IL-17F) (Zrioual et al., 2009; Goepfert et al., 2017; Robert and Miossec, 2024). IL-17F is encoded by a gene found on chromosome 6 in humans that is located near the IL-17A gene, reflecting their evolutionary and functional relationships (Giangrazi et al., 2024). IL-17F binds to the extracellular domains of both IL-17RA and IL-17RC, leading to the formation of a ternary complex. The interaction begins with IL-17F binding to IL-17RC, followed by the recruitment of IL-17RA to complete the signaling complex (Kuestner et al., 2007; Wright et al., 2008). Both IL-17RA and IL-17RC contribute to stabilizing the ligand‒receptor interaction, ensuring effective signal transduction (**Figure 2** and **Table 1**).

**Table 1:** Overview of IL-17 subtypes and associated receptors, effects on other cells, and relationships with disease

Name	Receptor	Main function	Association with disease
IL-17A, IL-17F and IL-17A-IL-17F heterodimers	IL-17RA	Pathogenesis of inflammatory disease	Psoriasis
	IL17RC	Neutrophil recruitment	Atopic eczema
		Host defense against extracellular pathogens and fungi	Multiple sclerosis
			Rheumatoid arthritis
			Psoriatic arthritis
			Chronic inflammatory
			intestinal diseases
			Inflammation with acute
			coronary syndrome
IL-17B	IL-17RB	Pro-inflammatory functions	Rheumatoid arthritis
IL-17C	IL-17RA	Pro-inflammatory functions	Psoriasis
	IL-17RE		Rheumatoid arthritis
			Chronic inflammatory intestinal diseases
IL-17D	CD93	Pro-inflammatory functions	Rheumatoid arthritis
IL-17E	IL17-RA	Th2 cell induction	Psoriasis
	IL17-RB	IL-17 and Th17 cell inhibition	Rheumatoid arthritis
			Chronic inflammatory intestinal diseases

CD93: Cluster of differentiation 93; IL-17A: interleukin-17A; IL-17B: interleukin-17B; IL-17C: interleukin-17C; IL-17D: interleukin-17D; IL-17E: interleukin-17E; IL-17F: interleukin-17F; IL-17RA: interleukin-17A receptor; IL-17RB: interleukin-17B receptor; IL-17RC: interleukin-17C receptor; IL-17RE: interleukin-17E receptor; Th17: helper T 17 cell.

## Molecular Pathways of the Interleukin-17 Family

**Figure 3:**
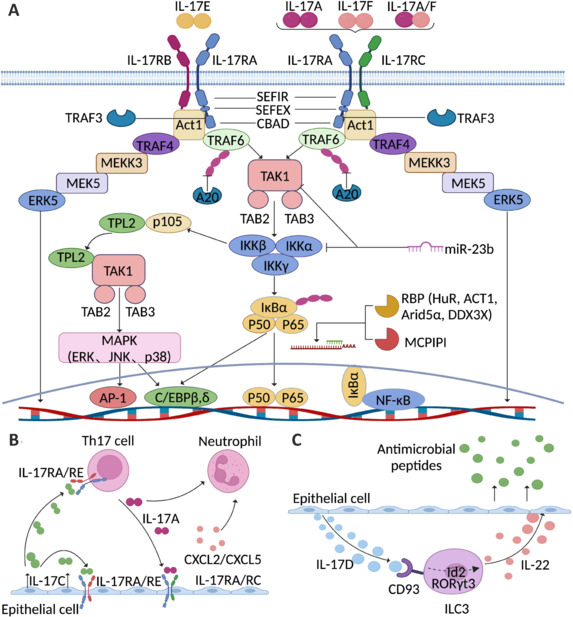
Molecular pathways of neuroimmune diseases mediated by IL-17. (A) IL-17 signaling starts with the binding of the IL-17A/A, IL-17A/F or IL-17F/F cytokines to their receptors (IL-17RA and IL-17RC) or the IL-17E/E cytokines to their receptors (IL-17RA and IL-17RB). Upon ligand binding, Act1 activates multiple independent signaling pathways that are mediated through different TRAF proteins. The activation of TRAF6 results in the triggering of the NF-κB, C/EBPβ, C/EBPδ and MAPK pathways. The IL-17R-Act1 complex also associates with MEKK3 and MEK5 via TRAF4, resulting in the activation of ERK5. Different classes of inhibitors, such as ubiquitinases (TRAF3), deubiquitinases (A20), RBPs (HuR, ACT1, Aridα, and DDX3X), endoribonucleases (MCPIP1/Regnase-1) and microRNAs (miR-23b), negatively regulate IL-17 signaling through various independent mechanisms. (B) Schematic of the proinflammatory mode of action of the IL-17C/RE axis. IL-17C is expressed mainly by epithelial cells. When the IL-17RA/RE receptor complex is expressed, both the epithelial cell itself and TH17 cells are targets of IL-17C. In addition to increasing the expression of IL17A in Th17 cells, IL-17C indirectly enhances the epithelial expression of chemokines that attract neutrophils, which ultimately causes a strong inflammatory reaction. (C) Interleukin-17D regulates group 3 innate lymphoid cell function through its receptor CD93. IL-17D is expressed primarily by colonic epithelial cells. CD93 is a functional receptor of IL-17D expressed on mature ILC3s. IL-17D deficiency leads to decreased CD93 activation, and CD93 deficiency inhibits ILC3 development and reduces ILC3 secretion of IL-22. Created with BioRender.com. Act1: Nuclear factor κB activator 1; CBAD: CCAAT/enhancer binding protein beta activation domain; CD93: cluster of differentiation 93; CXCL2: chemokine (C-X-C motif) ligand 2; CXCL2: chemokine (C-X-C motif) ligand 5; DDX3X: dead-box helicase 3 X-linked; ERK: extracellular signal-related kinase; HuR: heat-shock protein; IKK: inhibitor of kappa B kinase; IL-17: interleukin 17; IL-17A: interleukin-17A; IL-17C: interleukin-17C; IL-17D: interleukin-17D; IL-17E: interleukin-17E; IL-17F: interleukin-17F; IL-17RA: interleukin-17A receptor; IL-17RB: interleukin-17B receptor; IL-17RC: interleukin-17C receptor; IL-17RE: interleukin-17E receptor; IL-22: interleukin-22; ILC3: type 3 innate lymphoid cells; IκBα: inhibitor kappa B alpha; JNK: Janus kinase; MAPK: mitogen-activated protein kinase; MCPIPI: monocyte chemotactic protein-1-induced protein-1.

When IL-17A/A, IL-17A/F, or IL-17F/F interact with their receptors, IL-17RA and IL-17RC, the IL-17 signaling cascade, a fundamental process in modulating inflammatory and immune responses, is triggered (Hu et al., 2011). These receptors consist of two extracellular fibronectin-like domains that promote ligand interactions and an intracellular SEFIR domain, which is essential for triggering downstream signaling (Gaffen et al., 2008). Upon receptor activation, the SEFIR domains of IL-17RA and IL-17RC interact with the corresponding SEFIR sequence of Act1 to form a complex (Yang et al., 2008). This binding event is a critical phase in driving the cascade of cellular responses (**Figure 3**).

Act1 assembles and stimulates various TRAF proteins, triggering key intracellular signaling pathways, including the NF-κB, MAPK, and C/EBPβ and C/EBPδ transcription factors (Kanamori et al., 2002; Huang et al., 2007). These pathways drive the upregulation of various proinflammatory genes involved in immune cell recruitment, cytokine release, and tissue remodeling. NF-κB stimulation is essential for inducing the synthesis of proinflammatory cytokines such as IL-6, TNF-α, and IL-1β, which subsequently amplify the inflammatory response (Jeong et al., 2002; Zhu et al., 2015). Overall, the IL-17 pathway helps regulate the modulation of immune system activity across a range of inflammatory diseases, and its dysregulation may result in persistent inflammation and autoimmunity (Herjan et al., 2018; Douglas et al., 2023).

IL-17 can also stimulate the NF-κB pathway, subsequently triggering the expression of inflammatory transcription factors and stimulating the secretion of proinflammatory molecules (Shen et al., 2022; Li et al., 2023). Act1, which functions as an E3 ubiquitin ligase, aids in the interaction with TRAF6 and promotes its ubiquitination (Bie et al., 2021). Ubiquitinated TRAF6 then serves as a scaffold protein, enabling its assembly and the upregulation of TGF-β-activated kinase 1 and IκB kinase (IKK), thereby facilitating the nuclear import of NF-κB, which subsequently activates the transcription of inflammatory genes. Additionally, TRAF3 and TRAF4 bind with TRAF6. TRAF4 relies on the Act1 binding site, and its deletion potentiates IL-17-induced gene activation (Niu et al., 2013). Similarly, TRAF3 interacts with the CBAD domain located on IL-17RA, limiting IL-17-induced proinflammatory mediator expression by competing with TRAF6 (Yang et al., 2023a). Additionally, deubiquitination regulates NF-κB activity. Zinc finger protein A20 (Garg et al., 2013), a deubiquitinating enzyme recruited by the CBAD domain on IL-17RA, deubiquitinates TRAF6 by removing ubiquitin chains, thus inhibiting NF-κB activation through negative feedback (Deng et al., 2022). Similarly, ubiquitin-specific protease 25 deubiquitinates TRAF6, further suppressing IL-17 signaling.

In addition, IL-17 enhances the expression of C/EBPδ and C/EBPβ, two crucial transcription factors within the C/EBP family that are critical for the transcription of genes regulated by IL-17 (Shen et al., 2009). These transcription factors facilitate the activation of various proinflammatory genes, such as IL-6 and lipid carrier protein 2, by binding to specific promoter regions. Moreover, through its CBAD region, IL-17RA activates key signaling pathways that result in the phosphorylation of C/EBPβ. Specifically, the ERK1/2 and glycogen synthase kinase 3β pathways mediate threonine phosphorylation of C/EBPβ (Ding et al., 2017a). This phosphorylation modulates C/EBPβ activity, further enhancing the inflammatory cascade and stimulating the secretion of proinflammatory mediators such as IL-6. These mechanisms highlight the critical role of C/EBPδ and C/EBPβ in IL-17-driven inflammation and immune regulation (Karlsen et al., 2010).

IL-17 is also capable of initiating the MAPK pathway, which involves essential kinases such as ERK, p38, and c-Jun N-terminal kinase. In mouse models of autoimmune encephalomyelitis, the inhibition of p38 MAPK significantly reduces IL-17-induced pathological responses, whereas the deletion of a p38 MAPK inhibitor enhances IL-17 signaling, exacerbating inflammation (Zheng et al., 2024). Additionally, IL-17 stimulation prompts IKK to mediate p105 phosphorylation, which in turn activates both p38 and c-Jun N-terminal kinase, further contributing to the inflammatory and immune dysregulation associated with IL-17 signaling (Li et al., 2016).

In human keratinocytes, IL-17 initiates a signaling cascade through IL-17R, Act1, TRAF4, and MEKK3, resulting in the activation of ERK5 and increasing the expression of chemokines and antimicrobial peptides, thus establishing a proinflammatory environment (Wu et al., 2015). The IL-17-driven signaling cascade is further regulated by noncoding RNAs, such as miRNA-23b, which targets mRNAs encoding TGF-β-activated kinase 1-binding proteins (TAB2, TAB3) and IKKα, thereby inhibiting NF-κB activation (Zandman-Goddard et al., 2014). IL-17 suppresses the transcription of miRNA-23b, thereby amplifying its own signaling pathway.

**Table 2:** The function of IL-17 in neuroimmune and nonneuroimmune diseases

Disorder	Expression level	Source	Function of IL-17s	Reference
MS	Increased	CSF	Promote Th17 development and expansion	Sutton et al., 2009; Siffrin et al., 2010; Kang et al., 2013; Huang et al., 2022; Luo et al., 2023
		PBMCs	Impair the BBB	
			Induce IL-17R signaling	
			Increase expression of IL-6, and TNF-α	
			Inhabit OPCs development and viability	
			Promote neuronal damage and axonal injury	
NMOSD	Increased	Serum	Influence Breg cell’s function indirectly	Yokote et al., 2013; Zeka et al., 2016; Kim et al., 2021
		CSF	Synergistic role with AQP4 antibodies in NMOSD	
			Increase SAA level	
MG	Increased	Serum	Correlate with disease severity	Li et al., 2019b
		CSF		
GBS	Increased	Plasma	Unclear	Ma et al., 2022; Bnfaga et al., 2023
		Serum		
		CSF		
ADEM	Increased	CSF	Unclear	Van Steenhoven et al., 2023
Diabetes	Increased	Serum	Cause β-cell dysfunction	Tian et al., 2023
		PBMCs	Synergistic role with RORγ in T1D	
			Increase expression of IL-6, TNF-α and IL-1β	
			Promote the release of iNOS	
Psoriasis	Increased	Skin	Heighten keratinocyte growth	Glatt et al., 2018; Borowczyk et al., 2020; Cole et al., 2023
		Serum	Enhance neutrophil recruitment	
			Impair epidermal barrier integrity	
			Promote keratinocyte proliferation and differentiation	
HS	Increased	Skin	Recruit S100A8, S100A9 and NLRP3 to lesion	Matusiak et al., 2017
AD	Increased	Skin	Influence skin barrier function	Spidale et al., 2020
AA	Increased	Skin	Impair hair follicle regeneration	Loh et al., 2018
PRP	Increased	Serum	Involvement of the IL-23/IL-17 axis in the pathogenesis of PRP	Feldmeyer et al., 2017
SSc	Increased	Serum	Promote activation of the ERK1/2 pathway	Seki et al., 2024
RA	Increased	Joint synovium	Promote the synthesis of PGE2 and induce osteoclast differentiation	McDermott et al., 2016; Chen et al., 2023; Zhang et al., 2024
		PBMCs	Increase expression of CXCL1, CCL2, and CXCL5	
AS	Increased	Serum	Promote osteoclast differentiation	Rosenzweig et al., 2024
PsA	Increased	Synovial	Increase expression of MMP-1, MMP-9, and MMP-13	Cole et al., 2023
		membrane	Increase the expression of IL-8, and IL-6	
IBD	Increased	Intestinal epithelium	Increase the expression of CCL20	Hanna et al., 2022; Swedik et al., 2022
		Intestinal endothelium	Activate NF-κB, p38, and AP-1 signaling pathways	
			Serve a protective effect in certain contexts	

AA: Alopecia areata; AD: atopic dermatitis; ADEM: acute disseminated encephalomyelitis; AP-1: activating protein-1; AQP4: aquaporin 4; AS: ankylosing spondylitis; Breg: regulatory B; CCL2: chemokine (C-C motif) ligand 2; CCL20: chemokine (C-C motif) ligand 20; CXCL1: chemokine (C-X-C motif) ligand 1; CXCL5: chemokine (C-X-C motif) ligand 5; CSF: cerebrospinal fluid; ERK1/2: extracellular signal-related kinase 1/2; GBS: Guillain‒Barre syndrome; HS: Hidradenitis suppurativa; IBD: inflammatory bowel disease; IL-17: interleukin-17; IL-17R: interleukin-17 receptor; IL-1β: interleukin-1β; IL-23: interleukin-23; IL-6: interleukin-6; IL-8: interleukin-8; MG: myasthenia gravis; MMP-1: metalloproteinase-1; MMP-13: metalloproteinase-13; MMP-9: metalloproteinase-9; MS: multiple sclerosis; NF-κB: nuclear factor κB; NMOSD: neuromyelitis optica spectrum disorder; OPCs: oligodendrocyte precursors cells; PBMCs: peripheral blood mononuclear cell; PGE2: prostaglandin E2; PRP: pityriasis rubra pilaris; PsA: psoriaticarthritis; RA: rheumatoid arthritis; SSc: systemic sclerosis; T1D: type 1 diabetes; Th17: helper T cell 17; TNF-α: tumor necrosis factor α.

**Table 3:** Antibodies against IL-17, IL-17RA, CD20, CD19, IL-6R, IL-1β, and IL-23 approved for treatment

	Target	Stage	Organization	Adaptation
Secukinumab	IL-17A	Jan. 2015, FDA approved	Cosentyx	PsA, AS
		Jan. 2015, EMA approved		
		May. 2020, NMPA approved		
Lxekizumab	IL-17A	Mar. 2016, FDA approved	Eli Lilly	PsO, PsA, AS
		Apr. 2016, EMA approved		
		Aug. 2019, NMPA approved		
Netakimab	IL-17A	May. 2019, RMH approved	Biocad	PsO
Bimekizumab	IL-17A/IL-17F	Agu. 2021, EMA approved	UCB	PsO
Brodalumab	IL-17RA	Feb. 2017, FDA approved	AstraZeneca/Valeant	PsO
		Jul. 2017, EMA approved		
		Jun. 2020, NMPA approved		
Rituximab	CD20	Nov. 1997, FDA approved	Roche/Genentech	RA, GPA
		Jun. 1998, EMA approved		
		Apr. 2008, NMPA approved		
Ofatumumab	CD20	Jun. 2010, EMA approved	Novartis	MS, CLL, RA, GPA, PV
		Apr. 2014, FDA approved		
		Dec. 2021, NMPA approved		
Obinutuzumab	CD20	Nov. 2013, FDA approved	Roche/Genentech	LN, CLL, FL
		Jul. 2014, EMA approved		
Ocrelizumab	CD20	Mar. 2017, FDA approved	Roche/Genentech	RRMS, PPMS
Inebilizumab	CD19	Jun. 2020, FDA approved	AstraZeneca/Viela Bio	NMOSD
		Mar. 2022, NMPA approved		
Satralizumab	IL-6R	Jun. 2010, FDA approved	Roche/Genentech	NMOSD
Canakinumab	IL-1β	May. 2019, FDA approved	Novartis	SJIA, CAPS
Ustekinumab	IL-23	Jun. 2009, EMA approved	Johnson & Johnson	PsO
		Sep. 2009, FDA approved		
Guselkumab	IL-23	Jul. 2017, FDA approved	Johnson & Johnson	PsO, PsA
		Nov. 2017, EMA approved		
Tildrakizumab	IL-23	Mar. 2018, FDA approved	Merck & Co., Inc.	PsO
		Apr. 2019, EMA approved		
Risankizumab	IL-23	Apr. 2019, FDA approved	AbbVie	CD, PsO, PsA
		Apr. 2019, EMA approved		

AS: Ankylosing spondylitis; CAPS: cryopyrin-associated periodic syndrome; CD: Crohn's disease; CD19: Cluster of differentiation 19; CD20: Cluster of differentiation 20; CLL: chronic lymphocytic leukemia; FL: follicular lymphoma; GPA: granulomatosis with polyangiitis; IL-1β: interleukin-1β; IL-6R: interleukin-6 receptor; IL-17A: interleukin-17A; IL-17F: interleukin-17F; IL-17RA: interleukin-17A receptor; IL-23: interleukin-23; LN: lupus nephritis; MS: multiple sclerosis; NMOSD: neuromyelitis optica spectrum disorder; PsA: psoriatic arthritis; PsO: plaque psoriasis; PV: pemphigus vulgaris; PPMS: primary progressive multiple sclerosis; RA: rheumatoid arthritis; RRMS: relapsing-remitting multiple sclerosis; SJIA: systemic juvenile idiopathic arthritis.

RNA stability and decay are essential processes governed by elements in the 3′ untranslated region of mRNAs, which form interactions with various RNA-binding proteins (RBPs) (Zhu et al., 2012). IL-17 is crucial for regulating the function of these RBPs and determining whether mRNAs are stabilized or degraded. RBPs such as human antigen R, Act1, AT-rich interactive domain-containing protein 5a, and DEAD-box helicase 3-X-link contribute to mRNA stabilization, prolonging lifespan (Koshio et al., 2013; Sun et al., 2021; Herjan et al., 2022; Miyashita et al., 2022; Li et al., 2024a). Conversely, RBPs such as splicing factor 2 and monocyte chemotactic protein induced protein 1 (MCPIP1) drive MCPIP1 mRNA decay, reducing mRNA levels and subsequently limiting protein synthesis (Amatya et al., 2018).

CD93 has recently been recognized as a key receptor that interacts with IL-17D, and its involvement in regulating angiogenesis, inflammation, and tumor progression has been well documented (Samstein et al., 2019; Szczesny-Malysiak et al., 2020; Campagna et al., 2021). Nevertheless, the involvement of IL-17D in the downstream signaling pathways of CD93 remains insufficiently explored. CD93 is essential for regulating blood vessel formation, maintaining vascular integrity, and controlling permeability by interacting with crucial proteins such as insulin-like growth factor binding protein 7, multimerin 2 (MMRN2), VE-cadherin, and Moesin. Additionally, the C-type lectin-like domain of CD93 interacts with MMRN2, a component of the extracellular matrix. Similarly, the cytoplasmic tail of CD93 undergoes phosphorylation by Src kinase, facilitating the recruitment of the adaptor protein Cbl and the activation of Rho proteins (such as Rac1 and RhoA). This process promotes cytoskeletal rearrangement and enhances cell movement. Furthermore, soluble CD93 is an important angiogenic factor that can activate signaling pathways, including focal adhesion kinase, phosphatidylinositol 3-kinase (PI3K)/Akt/endothelial nitric oxide synthase, and ERK1/2, to promote endothelial cell migration and proliferation. Ni et al. (2022) identified a new mechanism through which IL-17D regulates inflammatory skin diseases by triggering the signaling cascade of CD93. In lesions associated with AD and psoriasis, elevated IL-17D activates the CD93-p38 MAPK/Akt/Smad2/3 pathway, resulting in the downregulation of DEAD-box helicase 5 (DDX5) expression in keratinocytes. This, in turn, affects the splicing of IL-36 receptor (IL-36R) pre-mRNA, promoting IL-36R expression and inhibiting the expression of soluble IL-36R. Soluble IL-36R reduces the inflammatory response in keratinocytes by competing with IL-36R for binding to the IL-36 ligand, thereby inhibiting IL-36/IL-36R signaling. This selectively amplifies the IL-36R-mediated inflammatory response, contributing to skin inflammation. Recently, IL-17D was identified for the first time as promoting atherosclerosis. These findings suggest that IL-17D accelerates the development of atherosclerotic plaques by inducing endothelial cell inflammation and ferroptosis through the CD93/miR-181a-5p/SLC7A11 signaling cascade. Furthermore, higher concentrations of IL-17D are strongly linked to a greater occurrence of cardiovascular adverse events (AEs), suggesting its potential as a therapeutic target and a circulating biomarker for heart-related conditions (Gu et al., 2024).

The existence of multiple nonredundant mechanisms regulating IL-17 signaling suggests that no single regulator can fully modulate IL-17 activity. Instead, the combined efforts of multiple regulators are needed, with each playing a distinct role at different points in the signaling cascade. Upon activation, such regulators may also modulate additional signaling networks, fine-tuning inflammatory responses. Biologically, maintaining an equilibrium between the detrimental and beneficial effects of IL-17 signaling is crucial.

## Cellular Source of Interleukin-17 Family

In 2003, researchers found that IL-23 activated T lymphocytes to release different cytokine profiles from known Th1 and Th2 cytokine profiles, which were identified as IL-17A (Aggarwal et al., 2003). In 2005, research revealed that IL-23 induces activated CD4^+^ T lymphocytes to express RORγt, a key regulator of gene expression, and identified this newly discovered cell type as Th17 (Langrish et al., 2005). As research advanced, it became clear that, beyond Th17 cells, various other cell types, including CD8^+^ T cells, γδ T cells, innate lymphoid cells (ILCs), natural killer cells, invariant natural killer T cells, mucosa-associated invariant T cells, mast cells, and Paneth cells, are capable of secreting IL-17A. Analogously, IL-17F is secreted by various immune populations akin to those that produce IL-17A. Initially identified within the human genome through its sequence homology to IL-17F, subsequent studies using intracellular staining with anti-IL-17F antibodies have confirmed that γδT and Th17 cells represent the principal contributors of IL-17F within the organism (Yang et al., 2008). 

**Figure 4:**
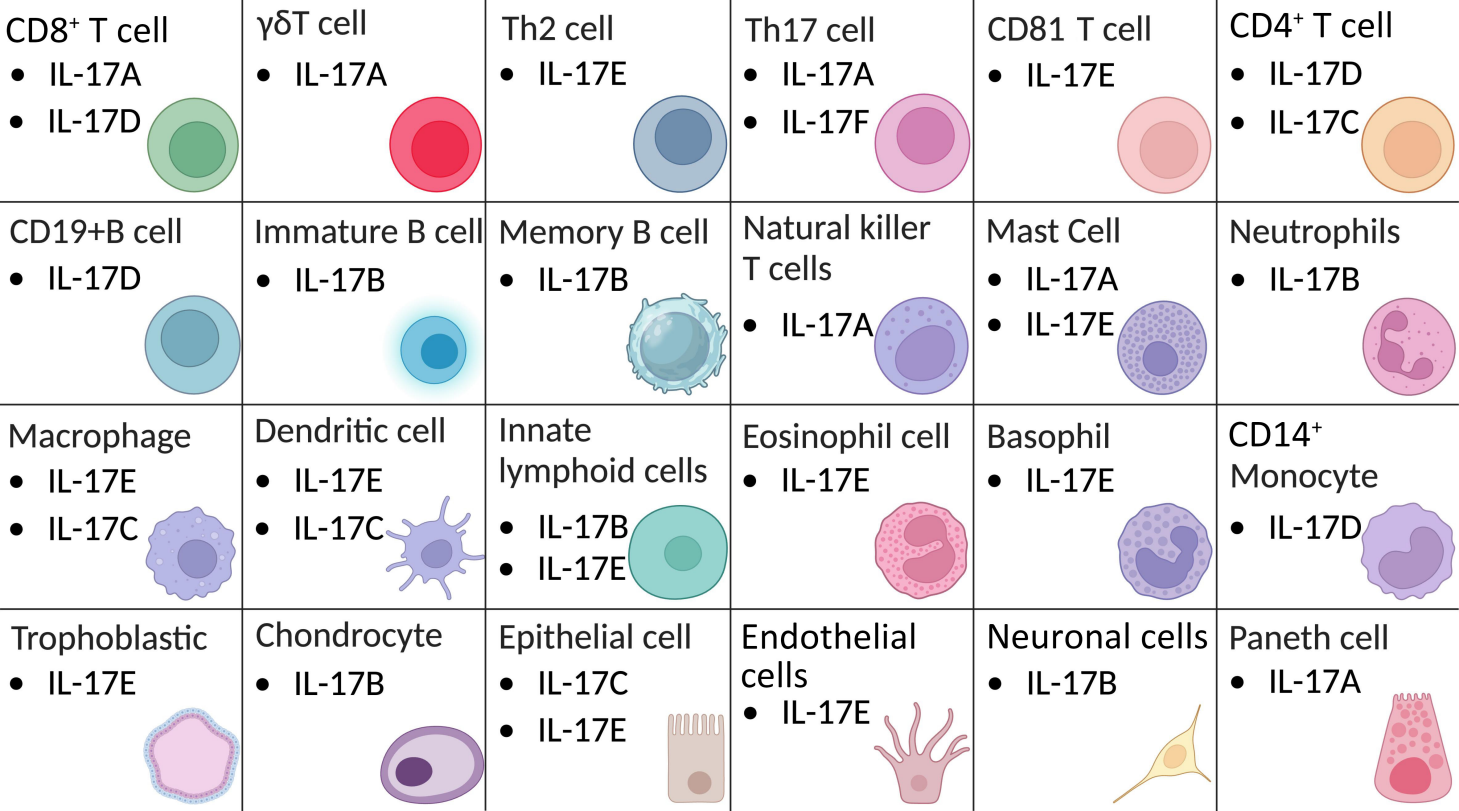
Major IL-17 family subtypes are expressed by different immune cell types. IL-17 family cytokines are secreted by diverse immune and nonimmune cells: IL-17A/F by CD8^+^ T cells, γδT cells, Th17 cells, natural killer T cells, mast cells and paneth cells; IL-17B by immature B cells, memory B cells, neutrophils, innate lymphoid cells, chondrocytes, neuronal cells; IL-17C by CD4^+^ T cells, macrophages, dendritic cells, epithelial cells; IL-17D by CD8^+^ T cells, CD4^+^ T cells, CD19^+^ B cells and CD14^+^ monocytes; and IL-17E by Th2 cells, CD81 T cells, mast cells, macrophages, dendritic cells, innate lymphoid cells, eosinophil cells, basophils, trophoblastic cells, epithellial cells, endothelial cells. Created with BioRender.com. CD4: Cluster of differentiation; CD8: cluster of differentiation 8; CD14: cluster of differentiation 14; CD19: cluster of differentiation 19; CD81: cluster of differentiation 81; IL-17A: interleukin-17A; IL-17B: interleukin-17B; IL-17C: interleukin-17C; IL-17D: interleukin-17D; IL-17E: interleukin-17E; IL-17F: interleukin-17F; ILC: innate lymphoid cell; Th2: helper T-cell 2; Th17: helper T-cell 17.

Li et al. (2000) discovered that Th17 cells fail to produce *IL-17B* mRNA or *IL-17C* mRNA. They then used RNA blotting to analyze *IL-17B* and *IL-17C* levels in different human tissues. A transcript of approximately 800 bp was detected in the pancreas, small intestine, and stomach, while a weaker signal was observed in the testes. However, no significant signal for IL-17C was detected in any tissues. Meanwhile, Shi et al. (2000) also observed strong signals around 1 kb in the spinal cord, testes, and small intestine tissues, weaker signals in the prostate, colon mucosal lining, ovary, and K562 cell line, and faint signals in the trachea, uterus, adrenal glands, substantia nigra, and fetal kidney. It was later discovered that IL-17B is abundantly present in chondrocytes and neurons. Al-Samadi et al. (2016) found that healthy colonic epithelial cells weakly express *IL-17B*, while certain connective tissue cells exhibit a strong expression of *IL-17B*. In moderately and poorly differentiated colon cancer cells, IL-17B tissue expression is elevated, and the number of IL-17B^+^ stromal cells in colorectal cancer was significantly higher than in the control group. Neutrophils have also been identified as cells capable of producing IL-17B (Kouri et al., 2014). In summary, IL-17B was identified in various cell types, including neuronal cells, mouse embryonic limb buds, chondrocytes, germinal center B cells, and both naive and memory B cells (Yamaguchi et al., 2007; Kokubu et al., 2008; Ferretti et al., 2016). Epithelial cells subsequently express IL-17C, with keratinocytes being the main producers in the context of psoriasis (Ramirez-Carrozzi et al., 2011; Al-Samadi et al., 2014). *IL-17C* exhibits high expression in healthy colonic surface epithelial cells and is also abundantly present in moderately differentiated colon cancer cells (Al-Samadi et al., 2016). Following *Candida albicans* infection in mouse kidney tissue, *IL-17C* was highly expressed in renal epithelial cells. However, no significant expression was observed in other infected tissues, such as the lungs, liver, and spleen (Sparber and LeibundGut-Landmann, 2015). Furthermore, alveolar epithelial, bronchial epithelial, and other respiratory epithelial cells have been identified as sources of IL-17C, which has a critical function in mediating these cells’ innate immune responses. Studies have shown that smooth muscle cells and leukocytes also express IL-17C, although it is primarily synthesized by epithelial cells (Hou et al., 2013). IL-17D is among the least studied members of the IL-17 family of cytokines (Ding et al., 2017b). Due to its high homology with IL-17B, IL-17D is abundant in various healthy human tissues, including skeletal muscle, adipose tissue, and the brain, heart, lungs, and pancreas (Starnes et al., 2002). In contrast, its expression is lower in tissues such as bone marrow, kidneys, liver, and spleen. Interestingly, low levels of IL-17D are also detected in quiescent CD4^+^ T cells and CD19^+^ B cells. Nevertheless, it is almost undetectable in the case of activated CD4^+^ T cells, all states of CD8^+^ T cells, CD14^+^ monocytes, and activated CD19^+^ B cells (Starnes et al., 2002). IL-17E was originally identified as a cytokine secreted by highly polarized Th2 cells, but its expression has since been detected in various other cell types. In the experimental autoimmune encephalomyelitis (EAE) mouse model, IL-17E expression in microglial cells of the central nervous system (CNS) increased nine-fold during disease onset, suggesting that IL-17E is crucial in modulating autoimmune disorders (Kleinschek et al., 2007). Similarly, brain endothelial cells have been demonstrated to produce IL-17E, which helps maintain the integrity of the blood–brain barrier (BBB) through a protein kinase Cepsilon-dependent mechanism (Sonobe et al., 2009). In addition, crosslinking with immunoglobulin E to stimulate bone marrow-derived mast cells induces elevated levels of *IL-17E* mRNA, with levels comparable to those of activated Th2 cells (Ikeda et al., 2003). In the lung lesion areas of rats treated with titanium dioxide (TiO_2_), macrophages displayed strong expression of IL-25 and IL-13. *In vitro*, stimulating alveolar macrophages with TiO_2_ triggered the production of IL-25 and IL-13 in a dose- and time-dependent manner (Kang et al., 2005). Furthermore, eosinophils and basophils were isolated from the blood of normal individuals and allergic subjects. Upon *in vitro* activation, both cell types secreted IL-17E. Notably, after immunoglobulin E crosslinking, basophils activated from allergic subjects produced approximately twice as much IL-25 as those from the normal control (Wang et al., 2007). Subsequent research has demonstrated that IL-17 occurs at different levels in immune cells, such as CD8^+^ T cells and dendritic cells, in addition to epithelial cells from different tissues (Senra et al., 2016; Borowczyk et al., 2020; Yuan et al., 2023; **Figure 4**).

**Figure 5:**
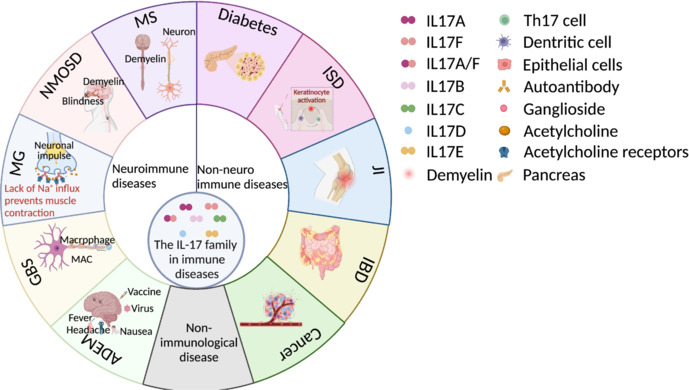
IL-17-related immune diseases. IL-17 cytokines mediate immune regulation, contributing to inflammation and pathology in autoimmune and immune-related diseases, including neuroimmune disorders (MS, NMOSD, MG, GBS, and ADEM) and nonneuroimmune conditions (diabetes, IBD, skin diseases, joint inflammation, and cancer). Created with BioRender.com. ADEM: Acute disseminated encephalomyelitis; GBS: Guillain–Barré syndrome; IBD: inflammatory bowel disease; IL-17A: interleukin-17A; IL-17B: interleukin-17B; IL-17C: interleukin-17C; IL-17D: interleukin-17D; IL-17E: interleukin-17E; IL-17F: interleukin-17F; ISD: inflammatory skin disease; JI: joint inflammation; MG: myasthenia gravis; MS: multiple sclerosis; NMOSD: neuromyelitis optica spectrum disorder; Th17: helper T-cell 17.

Immune diseases arise from an imbalance in immune regulation that impacts the body’s immune response (**Figure 5**). Th17 cells and IL-17 factors function like mediators in modulating immune function. The IL-17 family signals through its receptor, activating biological functions that regulate inflammation, infection, and other pathological processes, serving as a crucial regulatory mechanism in autoimmune diseases (**Table 2**).

## Physiological Functions of the Interleukin-17 Family

IL-17A is capable of inducing the production of proinflammatory cytokines, such as IL-6, TNF-α, and GM-CSF, as well as promoting the secretion of chemokines, including chemokine (C–X–C motif) ligand (CXCL)1 and CXCL8, to recruit and activate neutrophils, thereby exerting potent proinflammatory effects (Cheung et al., 2008; Lee et al., 2008; Sonderegger et al., 2008). In general, the proinflammatory role of IL-17A in autoimmune diseases is widely recognized. However, accumulating evidence has revealed that IL-17A also plays critical roles in immune responses, infection immunity, tissue regeneration, and neural repair. In PdgfraΔIl17ra conditional knockout mice, the absence of IL-17RA results in significantly reduced neutrophil recruitment and increased susceptibility to *Staphylococcus aureus* infection (Cavagnero et al., 2024). Similarly, in nonlethal intranasal infections with the *Streptococcus pyogenes* M1 strain in immunocompetent and IL-17-deficient mice, the lack of IL-17 led to the dissemination of *Streptococcus pyogenes* to lymphoid organs after a single intranasal exposure. Repeated intranasal exposures exacerbated this phenomenon (Mills et al., 2024). Epithelial repair requires cells to adapt to the hypoxic wound microenvironment through the evolutionarily conserved hypoxia-inducible factor (HIF). It has long been believed that hypoxia alone is adequate to autonomously trigger HIF1α-mediated metabolic remodeling. However, recent evidence suggests that IL-17A, which is produced by RORγt^+^ γδ T cells, is essential for optimal HIF1α activation in the epithelium at the wound margin. The IL-17A-HIF1α axis orchestrates metabolic reprogramming in injured epithelial cells, driving them toward a glycolytic program to promote epithelial cell migration (Konieczny et al., 2022). Wound healing typically progresses through three distinct stages: inflammation, proliferation, and remodeling/resolution (Boniakowski et al., 2017). During the inflammatory phase, neutrophils are the initial immune cells that migrate to the wound area. On the one hand, neutrophils collaborate with other immune cells to eliminate pathogens invading the wound, protecting the host from infection. In addition, proteases produced by neutrophils can cause tissue damage. This dual role highlights the seemingly paradoxical function of IL-17A in wound healing. Compared with wild-type mice, IL-17A knockout enhances fibroblast differentiation and collagen deposition and accelerates wound closure. Administration of recombinant IL-17A leads to neutrophil recruitment, which impairs the wound healing process (Boniakowski et al., 2017). In addition, Enamorado et al. (2023) reported that commensal microbes in skin tissue can induce the upregulation of genes related to tissue repair, wound healing, and interactions with neurons in Th17 cells. This led to the hypothesis and subsequent validation that IL-17A secreted by Th17 cells may promote the regeneration of peripheral neurons. These findings identify a potential therapeutic target for enhancing nerve repair after injury or limiting neuropathy in the context of diabetes and chemotherapy.

Unlike IL-17A, which is expressed predominantly by Th17 cells, IL-17B is prevalent in multiple tissues. IL-17B does not exert strong proinflammatory effects associated with IL-17A and is thought to contribute significantly to embryonic formation, tissue restoration, and tumor advancement. Previous studies have reported that IL-17B can be detected on day 11 of embryonic development in mice, reaches its peak on day 15, and then begins to decrease after day 17 (Moore et al., 2002). Similarly, *IL-17B* mRNA was most highly expressed in the limb buds of mouse embryos at 14.5 days postcoitus and decreased to low levels by 19.5 days postcoitus. A previous study has shown that in small hepatocyte-like progenitor cells (SHPCs) of livers transplanted with Thy1^+^ cells, *IL-17RB* expression is notably elevated (You et al., 2005). Additionally, on day 14 after transplantation, both the quantity and size of SHPC clusters in the liver increased. Extracellular vesicles derived from the culture medium of Thy1^+^ cells triggered the expression of *IL-17B* and *IL-25* in sinusoidal endothelial cells and Kupffer cells. IL-17B stimulation promoted the growth of SHPCs in culture, suggesting that Thy1^+^ EVs, by targeting sinusoidal endothelial cells, Kupffer cells, and SHPCs, coordinate the IL-17RB signaling pathway to facilitate liver regeneration (Ichinohe et al., 2017). Li et al. (2021a) explored the metabolic cooperation between pancreatic stellate cells (PSCs) and tumor cells in pancreatic ductal adenocarcinoma, with an emphasis on the IL-17B/IL-17RB signaling pathway. Tumor-derived IL-17B, carried by extracellular vesicles, activates PSCs and increases the level of IL-17RB. Activated PSCs exhibit enhanced oxidative phosphorylation, reduced mitochondrial turnover, and stimulated tumor cells through a positive feedback mechanism, leading to elevated oxidative phosphorylation and suppressed glycolysis in tumor cells. Additionally, overexpression of *IL-17RB* in PSCs promoted tumor development in mice, further validating the role of this signaling pathway in tumor progression.

IL-17C is commonly expressed on mucosal and barrier surfaces and plays a significant role in the barrier functions of local tissues (Conti et al., 2015). IL-17C binds to the IL-17RA/RE receptor complex, stimulating epithelial cells to secrete proinflammatory cytokines, chemokines, and antimicrobial peptides. While IL-17C promotes inflammation in an imiquimod-induced skin inflammation model, it has a protective effect on dextran sulfate sodium-induced colitis (Ramirez-Carrozzi et al., 2011). Additionally, Vandeghinste et al. (2018) used an anti-IL-17C reference antibody (MOR106) to inhibit the interaction of IL-17C with its receptor in both an IL-23-induced psoriasis mouse model and an MC903-induced AD mouse model. They reported a significant reduction in skin inflammation in both models, along with suppressed expression of thymic stromal lymphopoietin (TSLP) and IL-33 in the AD mouse model. Increasing evidence suggests that IL-17C could serve as a potential target for treating autoimmune skin diseases.

While research on IL-17D is still limited, existing studies suggest that IL-17D is involved in tumor progression and anti-infection responses. Saddawi-Konefka et al. (2016) reported that the transcription factor Nrf2 (nuclear factor erythroid 2–related factor 2) promotes IL-17D expression, and this Nrf2/IL-17D axis facilitates the recruitment of natural killer cells, resulting in the regression of established tumors. These findings provide a potential approach for cancer treatment. Infection with group A Streptococcus in both wild-type and IL-17D-deficient mice (*il17d*^–/–^) revealed that *IL-17D* knockout mice experienced greater weight loss, reduced survival rates, and a greater bacterial load in the kidneys and peritoneal cavity after infection. Notably, in nonimmune cells, group A Streptococcus-mediated induction of IL-17D requires live bacteria, suggesting that the induction of IL-17D involves mechanisms beyond the mere recognition of pathogen-associated molecular patterns (Washington et al., 2020).

Surprisingly, recent reports have shown that IL-17D can promote pathogen infection by inhibiting the activity of CD8^+^ T cells. Compared with wild-type mice, IL-17D-deficient mice exhibit increased resistance to Listeria infection but also alleviated weight loss during influenza A virus infection and reduced pathogen burden (Lee et al., 2019). A better grasp of the roles and mechanisms of IL-17D requires further research in the future.

IL-17E, also known as IL-25, is a significant member of the IL-17 cytokine family and has distinct immunoregulatory functions. Compared with other family members, its primary role is skewed toward driving Th2 cell immune responses and combating parasitic infections (Fallon et al., 2006; Owyang et al., 2006; Oliphant et al., 2011). It also plays a key role in the onset of inflammation and allergic diseases. Specialized tuft cells, which are rare epithelial cells present in mucosal tissues such as the intestine, respiratory tract, biliary tract, and taste organs, play a central role in defending the body against parasitic infections (von Moltke et al., 2016; Rajeev et al., 2021). Tuft cells serve as the primary source of IL-25 in the intestine. Following helminth infection, IL-25 released by tuft cells activates type 2 innate lymphoid cells (ILC2s), leading to the secretion of IL-13, which initiates a defense reaction to combat parasites. The tuft cell-ILC2 circuit enhances intestinal remodeling and drives Th2-type immunity to expel parasites (Campillo Poveda et al., 2023). In addition, tuft cells have been identified as capable of secreting acetylcholine. From a functional perspective, IL-13-amplified tuft cells secrete acetylcholine into the intestinal lumen, which directly reduces the reproductive capacity of helminths via their expression of muscarinic acetylcholine receptors (Ndjim et al., 2024). In allergic inflammation, such as asthma, IL-17E induces epithelial cells and eosinophils to secrete chemokines, such as CCL11 and CCL17, attracting inflammatory cells to the airways, enhancing airway smooth muscle cell reactivity and contributing to airway remodeling (Margelidon-Cozzolino et al., 2022). TSLP and IL-33 have been shown to target various cell types, similar to IL-25, to promote type 2 cytokine responses. In medium-chain triglyceride/egg white-induced food allergy mouse models, IL-25, IL-33, and TSLP are indispensable. Blocking any one of these cytokines with monoclonal antibodies inhibits the development of food allergy, while the combined use of all three cytokines has the strongest suppressive effect on food allergy (Khodoun et al., 2018). Neutralizing or blocking these cytokines may represent a potential therapeutic strategy for treating established food allergies in humans. Similarly, in ADs, IL-17E expression is upregulated, enhancing the secretion of ILC2- and Th2-type cytokines. By stimulating keratinocytes, IL-17E also induces the secretion of TSLP and IL-33, further amplifying allergic inflammation (Nakajima et al., 2021; Stanbery et al., 2022).

IL-17F, first identified in 2001, shares a high degree of sequence homology (**~**50%) and structural similarity with IL-17A. It can be secreted either as a homodimer or as a heterodimer with IL-17A, signaling through the IL-17RA/IL-17RC receptor complex. This pathway drives the production of proinflammatory cytokines and chemokines across various cell types. IL-17F, in conjunction with IL-17A, contributes significantly to the onset and progression of various chronic inflammatory and autoimmune diseases (Chang and Dong, 2009). A previous study demonstrated the presence of not only IL-17A but also IL-17F in the psoriatic lesions and synovial tissues of patients with psoriatic arthritis (PsA) (Navarro-Compán et al., 2023). When synovial cells derived from PsA patients and primary normal human dermal fibroblasts were stimulated with supernatants from polyclonal human Th17 cells, blocking IL-17F alone did not significantly affect the release of proinflammatory cytokines such as IL-8 and IL-6. However, compared with blocking IL-17A alone, dual blockade of IL-17A and IL-17F reduced the secretion of IL-8 and IL-6 by 28% and 42%, respectively, while also significantly enhancing the suppression of neutrophil migration (Glatt et al., 2018). However, in psoriasis, IL-17A and IL-17F exhibit distinct gene expression profiles and regulatory mechanisms in Th17 cells. Among them, IL-17F is more strongly associated with cell proliferation. Despite these differences, both cytokines play important roles in driving inflammation, with IL-17F being linked to steroid-resistant inflammation. Furthermore, this study highlights the plasticity of Th17 cells, where IL-17F-producing cells can transition to produce IL-17A, and vice versa. This plasticity, along with the differential regulation by cytokines such as STAT3 and STAT5, highlights the complexity of IL-17 signaling in psoriasis (Cole et al., 2023).

## Function of Interleukin-17 in Immune Diseases

The interplay and regulation between the nervous and immune systems are fundamental to the normal functioning of the human body. All immune organs are innervated, and the immune system contributes to immune surveillance within the central nervous system (CNS).

### Multiple sclerosis

MS is an autoimmune disease characterized by inflammatory demyelinating lesions in the white matter of the CNS. It leads to damage of nerve fibers in the brain and spinal cord, affecting cognitive, emotional, motor, sensory, and visual functions (Zhang et al., 2024b). The precise mechanisms driving the pathogenesis of MS are yet to be clarified. However, the pivotal role of Th17 cells and IL-17 in the development of MS is widely acknowledged. In MS patients, the migration of Th17 cells into the CNS involves a multi-step process governed by intricate molecular interactions (Kebir et al., 2007). Th17 cells specifically express the chemokine receptor chemokine C-C-motif receptor 6 (CCR6), which binds to its cognate ligand CCL20 produced by BBB endothelial cells and choroid plexus epithelial cells under inflammatory conditions (Ikawa et al., 2021). This CCR6/CCL20 axis serves as a critical chemotactic signal that directs Th17 cell trafficking toward CNS compartments (Williams et al., 2020). Upon approaching the BBB, Th17 cells engage in dynamic crosstalk with endothelial cells through adhesion molecules such as α4β1 integrin (VLA-4) and its endothelial counterpart vascular cell adhesion molecule 1, facilitating firm adhesion and subsequent transendothelial migration (Balasa et al., 2020). Notably, IL-17 and IL-22 secreted by Th17 cells may further enhance BBB permeability by downregulating tight junction proteins such as claudin-5 and occludin. Once within the CNS parenchyma, these cells preferentially accumulate in active demyelinating lesions through synergistic interactions between locally produced CCL2 and CXCL12 and their corresponding receptors.

A recent single-cell study has identified a distinct “CNS-homing” Th17 subset co-expressing CCR6 and GM-CSF, suggesting functional heterogeneity in their pathogenic potential. This coordinated multi-molecular process highlights potential therapeutic targets, as evidenced by preclinical models demonstrating reduced neuroinflammation following CCR6 blockade or CCL20 neutralization (Restorick et al., 2017). A heightened percentage of Th17 cells and augmented *IL-17* mRNA levels were observed in brain lesion areas of MS patients, correlating positively with disease activity. IL-17 expression was observed in 79% of T cells in acute lesions and 73% of the active regions of chronically active lesions. Conversely, only 17% of T cells were detected in inactive lesions and 7% in lymph nodes (Moser et al., 2020). Furthermore, increased IL-17A concentrations exist in the cerebrospinal fluid (CSF) of patients clinically labeled as having relapsing-remitting MS (Ghezzi et al., 2020). In the EAE model, the most widely used animal model of MS, Th17 cells are identified as a key T cell population responsible for mediating disease pathology (Duesman et al., 2023; Xie et al., 2023). Evidence also suggests that Th1 cells can contribute to EAE pathology, but Th17 cell-induced EAE is more severe than Th1 cell-induced EAE (Loos et al., 2020). A study has revealed that CD161^hi^ CCR6 γδ T cells, which are prone to producing IL-17, exhibit an elevated presence within the CSF of individuals diagnosed with MS (Schirmer et al., 2013). A substantial number of γδT17 cells are present in the CNS of EAE mouse models, particularly at disease onset, and their depletion prevents disease development (Sutton et al., 2009). Some research indicates that the pathogenic cells mediating EAE CNS pathology are GM-CSF^+^ interferon-gamma (IFN-γ)^+^ C–X–C chemokine receptor type 6 (CXCR6)^+^ Th17 cells, which possess the transcription factor 1 (TCF1)^+^ and IL-17^+^, and the SLAMF6^+^ characteristics of stem-like T cells, and are sustained through the microbiota (Schnell et al., 2021).

In the EAE model, IL-17 impairs the BBB by promoting oxidative stress in endothelial cells and decreasing the levels of occludin, a critical component of tight junctions (Siffrin et al., 2010). Additionally, concurrently stimulating the human cortical microvessels endothelial cells/D3 BBB cells with IL-17A and IL-6 compromises cell adhesion junctions and monolayer integrity (Setiadi et al., 2019). IL-17 has been demonstrated to facilitate the onset of EAE through its targeting of resident CNS cells, including endothelial cells, astrocytes, microglia, NG2^+^ oligodendrocyte precursors cells (OPCs), and resident neuroectodermal cells (Luo et al., 2023). Human and mouse astrocytes exhibit the IL-17F receptor (Kang et al., 2013), and, upon activation, release cytokines and chemokines, such as IL-6, TNF-α, CXCL2, CXCL9, CXCL10, CXCL11, and CCL2, which influence immune cell infiltration and neuroinflammation (Lepennetier et al., 2019; Jiang et al., 2023). Human astrocytes specifically produce IL-6, which supports CD4^+^ naive cell differentiation into Th17 cells (Huang et al., 2022). Research has demonstrated that suppressing Act1 in astrocytes—thereby impeding the IL-17 pathway and consequently hindering the expression of associated chemokines—can diminish Th17 infiltration and mitigate the severity of ongoing EAE (Yan et al., 2012).

Zimmermann et al. (2018) used the copper ketone (CPZ) model in mice with astrocytes that had been genetically engineered to produce IL-17 in the CNS. CPZ-induced demyelination in mice was characterized by T-cell-independent mechanisms of myelin loss and glial responses. The study revealed that the genetically engineered production of IL-17 resulted in a more severe disease course in mice. Additionally, the presence of IL-17 during CPZ treatment resulted in enhanced accumulation of activated microglia and prolonged microglial proliferation during myelin degeneration. IL-17-stimulated microglia produce inflammatory mediators, and, in combination with Th1/Th17 cells, they generate IL-1β, IL-6, and TNF-α, promoting the enhanced formation of Th17 cell development, intensifying the severity of neuroinflammation and contributing to neuronal injury (Liu et al., 2019). IL-17 also impacts OPCs, inhibiting their development and viability *in vitro*. OPCs can differentiate into oligodendrocytes and produce myelin; IL-17 stimulation induces NOTCH1 activation in OPCs, and NOTCH1 (neurogenic locus notch homolog protein 1) signaling leads to defective myelin regeneration by impairing OPC differentiation (Wang et al., 2017). This study found that stimulating IL-17 increased Kv1.3 (voltage-gated K^+^(Kv) channel 1.3) expression in both *in vitro* OPC cultures and lysophosphatidylcholine-induced demyelination mouse models. This increase led to decreased AKT activation, the inhibition of OPC proliferation, and myelin damage (Li et al., 2021b).

Neuronal cells also harbor IL-17Rs, suggesting that IL-17 might contribute to causing neuronal damage (Arenas et al., 2024). Confocal, electron, and intravital microscopy have demonstrated direct contact between Th17 cells specific to myelin oligodendrocyte glycoprotein and neurons within areas of myelin loss, resembling immunological synapses (Siffrin et al., 2010). This interaction suggests that Th17 cells are crucial in driving neuronal damage and contributing to axonal injury. IL-17A expression has been shown to impair hippocampal long-term potentiation in the EAE model. IL-17RA has been found to be highly expressed in the CA1 region of the hippocampus, and the exposure to IL-17A has been shown to disrupt hippocampal long-term potentiation in a dose-dependent manner through the activation of its receptor and p38 MAPK, suggesting a potential involvement of the IL-17 axis in IL-17A expression has been shown to impair hippocampal long-term potentiation MS-associated cognitive impairment (Di Filippo et al., 2021).

The dynamic equilibrium between Th17 cells and regulatory T cells (Treg) constitutes one of the core mechanisms for maintaining immune homeostasis. In contrast to the proinflammatory Th17 cells, Treg cells suppress effector T cell activity by releasing anti-inflammatory factors such as IL-10, TGF-β, and IL-35, thereby sustaining peripheral immune tolerance. Under physiological conditions, the Th17/Treg balance is coordinately regulated by transcription factors RORγt and forkhead box protein 3, cytokines IL-6 and TGF-β, and metabolic signaling pathways, including the mTOR pathway (Guo et al., 2023). However, under pathological conditions, impaired Treg functionality or the resistance of Th17 cells to Treg-mediated suppression disrupts this equilibrium, leading to exacerbated inflammatory CNS damage. This imbalance arises from dysregulated interactions between proinflammatory and immunosuppressive mechanisms, ultimately compromising immune tolerance and amplifying neuroinflammatory pathology (Prado et al., 2021). In the EAE model, β-Elemene administration significantly reduced the expression of *IL-6*, *IL-23*, and *RORγt* in the spinal cords of mice while upregulating forkhead box protein 3 expression. This inhibition of Th17 cell differentiation and development, coupled with the promotion of Treg cell expansion, reduced inflammatory cell infiltration and axonal damage in the CNS, thereby alleviating EAE symptoms (Zhang et al., 2011).

Similar phenomena were observed following treatment with salmon proteoglycan (Sashinami et al., 2012). Mesenchymal stem cells (MSCs), owing to their potent immunomodulatory capabilities and low immunogenicity, have been widely applied in treating MS (Hu et al., 2024a). Studies demonstrate that MSCs regulate Th17/Treg homeostasis through pathways such as extracellular vesicle secretion, metabolic reprogramming, mitochondrial transfer, and autophagy, thereby maintaining immune self-tolerance and stabilizing immune equilibrium, ultimately mitigating neuroinflammation and demyelination (Luz-Crawford et al., 2019; Mendt et al., 2021; Menshikov et al., 2021; Yang et al., 2021). A recent study developed a Th17 cell-based “Trojan horse” nanocapsule system that exploits the ability of Th17 cells to penetrate the BBB and infiltrate CNS inflammatory sites. This system enables reactive oxygen species (ROS)-responsive release of the drug aminooxyacetic acid, inducing the in situ conversion of Th17 cells into Treg cells, effectively restoring immune balance and alleviating neuroinflammation and demyelination in an MS mouse model. This innovative approach provides a novel strategy for targeted therapy in autoimmune diseases (Shi et al., 2023).

Th17 cells have been identified as pivotal in the inflammatory processes central to the development of MS, making them critical therapeutic targets. Secukinumab, an antibody targeting IL-17A, has been evaluated in phase II trials, with magnetic resonance imaging results demonstrating a reduction in MS-related brain lesions following treatment (Havrdová et al., 2016). Secukinumab exerts anti-inflammatory, neuroprotective, and antioxidant effects in CPZ models (Abdel-Maged et al., 2020).

### Neuromyelitis optica spectrum disorder

Neuromyelitis optica spectrum disorder (NMOSD) is classified as an immune-mediated disorder marked by CNS inflammation driven primarily by B cells. Recent findings indicate that T cell-mediated immune responses, especially those involving the Th17 subset, may also contribute significantly to NMOSD pathogenesis (Kim et al., 2021; Xu et al., 2025).

IL-17 is present at increased concentrations in both CSF and serum during relapses in NMOSD patients (Huang et al., 2022), and its increased expression in active NMOSD lesions may explain the neutrophil accumulation, as seen in EAE models (Hertwig et al., 2016). Variants in IL-17-related genes are linked to aquaporin 4 (AQP4) antibody-positive NMOSD, suggesting a role of IL-17 in disease pathogenesis. Th17 cells are found in active NMOSD lesions, and there is an increased frequency of AQP4p61-80-specific Th17 cells targeting immunodominant epitopes (Guo et al., 2022). Although B cell depletion therapy has demonstrated efficacy, NMOSD IgG alone does not induce lesions in mice, while co-transfer with myelin-specific T cells leads to CNS inflammation, suggesting a cooperative role between T cells and AQP4 antibodies in NMOSD (Zeka et al., 2016). Serum amyloid A levels in NMOSD indicate that the increase in serum amyloid A is more associated with Th17 cell responses than systemic autoimmune reactions (Yokote et al., 2013). Additionally, two studies have demonstrated elevated concentrations of IL-6, IL-17, IL-21, and IL-1β in both CSF and the peripheral blood of NMOSD patients (Correale and Fiol, 2004; Min et al., 2012). The accumulated findings from various studies suggest that B cell-dependent inflammatory response alone might not account for NMOSD-like lesions, with Th17 cells potentially playing a significant role.

### Myasthenia gravis

Myasthenia gravis (MG) is an autoimmune neuromuscular disease driven primarily by acetylcholine receptor autoantibodies, muscle-specific tyrosine kinase (MuSK) autoantibodies, and low-density lipoprotein receptor-associated protein 4 autoantibodies, which result in damage to the postsynaptic membrane at the neuromuscular junction (Pevzner et al., 2012). Cytokines manufactured by Th17 cells, with particular emphasis on IL-17, are crucial in driving chronic inflammation at the neuromuscular junction in MG, contributing to its immunopathogenesis. The upregulation of IL-17 has been observed in the serum and CSF of MG patients, and this upregulation is correlated with an elevation in disease severity and clinical parameters (Li et al., 2019b). Th17 cells secrete a range of cytokines that activate immune cells like neutrophils and facilitate their migration toward targeted tissues in anti-MuSK antibody-positive MG. IL-17A and IL-21 levels showed a marked elevation in peripheral blood mononuclear cells, reflecting a heightened inflammatory state associated with the disease (Li et al., 2019b). Nevertheless, the exact biological pathways through which IL-17/Th17 cells exert their effects on this subset of MG are yet to be completely elucidated. The current study demonstrates that the attenuation of IL-17 results in a reduction in the severity of experimental autoimmune MG (Aguilo-Seara et al., 2017). Evidence suggests that the activation of the IL-23/Th17 axis leads to thymic hyperplasia, further supporting its involvement in MG pathogenesis (Debnath et al., 2018). Additionally, the relationship between IL-17A and accompanying anti-acetylcholine receptor antibody titers show the potential of IL-17 as a biomarker for MG, suggesting that modulating the IL-17/Th17 axis could offer a promising medical strategy. Given the complexity of Th17-mediated inflammation, subsequent investigations should concentrate on elucidating the detailed mechanisms and therapeutic implications of modulating the IL-17 pathway in MG (Sun et al., 2019; Ashida et al., 2021; Cebi et al., 2023).

### Guillain–Barre syndrome

Guillain–Barre syndrome (GBS) is an immune dysfunction condition that targets the peripherally located neural structures, frequently following an infection (Berciano et al., 2017). It is characterized by the generation of autoantibodies, which trigger the complement cascade, coupled with T cell responses against gangliosides (Ilyas et al., 1988; Wiwanitkit, 2010). Recent research suggests that GBS may be linked to Th17 cell dysregulation, leading to a rise in the levels of proinflammatory cytokines, particularly those in the IL-17 family, which drive autoimmune responses. According to Bnfaga et al. (2023), patients with GBS show elevated peripheral Th17 cell counts along with increased serum IL-17 levels. Notably, IL-17 levels are observed in higher amounts in both plasma and CSF during the acute phase of GBS, correlating with disability scores (Li et al., 2012). Recent work has provided evidence suggesting that IL-17 levels are elevated during the acute phase compared to the recovery phase, indicating that IL-17 can serve as a biomarker for tracking disease advancement (Jaramillo-Valverde et al., 2019). Additionally, a gene polymorphism (Glu126Gly) in the *IL-17* gene is tightly connected to a significantly elevated probability of GBS. Elevated IL-17 levels in the acute phase have also been linked to treatment responses (Paradowska-Gorycka et al., 2015). Plasma and CSF IL-17A levels decrease significantly in GBS individuals receiving intravenous immunoglobulin therapy (Ma et al., 2022). The involvement of the final elements of the complement cascade in GBS is well-established, highlighting the functional importance of IL-17 in GBS pathology.

### Acute disseminated encephalomyelitis

Acute disseminated encephalomyelitis (ADEM) is an idiopathic condition characterized by demyelination in the CNS, most frequently observed in children but capable of occurring at any age (Deery et al., 2010; Esposito et al., 2015). Current evidence suggests that ADEM involves auto-reactive T cell activation, leading to a temporary autoimmune reaction targeting myelin or other self-antigens (Nazerai et al., 2020). One study demonstrated that myelin-reactive T cells in ADEM patients were approximately 10 times higher than in healthy individuals. Additionally, CD3^+^ T cells secreting IFN-γ increased over several days, whereas CD4^+^ T cells secreting IL-17F showed no significant changes over the same period. A study reported no variation in CSF cytokine concentrations between EVE and anti-N-methyl-D-aspartate receptor encephalitis (Van Steenhoven et al., 2023). However, ADEM patients exhibited significantly elevated concentrations of Th17-related cytokines (IL-17F and IL-21) and Th2-associated cytokines/chemokines (IL-4 and CCL17) (Ma et al., 2022).

### Autoimmune encephalitis

Autoimmune encephalitis (AE) is a condition whereby the immune system incorrectly identifies self-antigens in the CNS as foreign antigens (Uy et al., 2021). This triggers an immune response that destroys these self-antigens. Although AE is an antibody-mediated disease, investigations have demonstrated the potential of IL-17A levels in CSF as a prognostic marker for disease severity in instances of non-N-methyl-D-aspartate receptor AE (Levraut et al., 2021). A recent study revealed a substantial accumulation of Th17 cells in the CSF of patients diagnosed with anti-N-methyl-D-aspartate receptor encephalitis (Zeng et al., 2022). This accumulation was accompanied by a notable increase in IL-17 concentration compared to the control group. Additionally, higher IL-17 levels were strongly linked to reduced treatment response and an increased likelihood of relapses in patients with anti-N-methyl-D-aspartate receptor encephalitis. Consequently, IL-17 may assist in IL-17 pathogenesis and progression by inducing inflammatory responses and immune-mediated injury. IL-17 levels in the CSF have the potential to serve as an indicator of disease progression and prognosis.

### Diabetes

Diabetes, a disease affecting people worldwide regardless of age or sex, has multiple and complex drivers (Ciarambino et al., 2022; Karunakaran, 2025; Ntetsika et al., 2025). Lately, increasing attention has been paid to the involvement of IL-17/Th17 cells in the pathogenesis of both type 1 (T1D) and type 2 (T2D) diabetes (Abdel-Moneim et al., 2018). Through IL-17A secretion, Th17 cells exhibit a crucial function in the inflammatory processes that underlie β-cell dysfunction in T1D (Qiu et al., 2021). When murine insulinoma cells were treated with a combination of IL-17A and streptozotocin, inflammatory cytokines, including TNF-α, IL-1β, IFN-γ, and inducible nitric oxide synthase, were significantly upregulated, aggravating β-cell damage by promoting oxidative stress and apoptosis while suppressing insulin secretion. Concurrently, IL-17A enhanced the expression of *IL-17RA*, thereby activating downstream signaling pathways such as NF-κB. This activation further induced the expression of pro-apoptotic genes including Fas, ultimately leading to β-cell death (Lamhamedi-Cherradi et al., 2003; Patel and Santani, 2009). RORγt, the master transcription factor for IL-17A, has been shown to be pivotal in driving the progression of T1D (Finucane et al., 2014; Ozgur et al., 2023). A study has revealed that during the establishment of the diabetes model in C57BL/6 mice, intraperitoneal administration of ethyl acetate extract of oregano significantly reduced the incidence of diabetes. Ethyl acetate extract of oregano treatment decreased the expression of key transcription factors T-bet and RORγT, reducing the number of CD4^+^ T cells and their subsets (including Th1 and Th17) (Vujicic et al., 2016). Recent research has also found that the level of IL-17A in the pancreas of RORγ-deficient mice is significantly lower than in streptozotocin-treated wild-type mice. This reduction leads to a significant decrease in pancreatic islet inflammatory infiltration, effectively protecting the function of β-cells and maintaining the function of glucagon-secreting α-cells (Tian et al., 2023). The potential of targeting the IL-17A/RORγt axis is further underscored by the novel RORγt inverse agonist, panaxadiol, demonstrated to suppress IL-17A production and alleviate inflammation, offering a promising therapeutic approach for T1D (Tian et al., 2023).

The role of IL-17 in T2D is pivotal due to its proinflammatory effects, which drive insulin resistance and β-cell dysfunction (Færch et al., 2015). IL-17 production is stimulated by leptin and macrophage migration inhibitory factor, which in turn enhance the concentrations found in IL-6, TNF-α, and IL-1β cytokines recognized for their role in amplifying systemic inflammation and contributing to insulin resistance (Finucane et al., 2014). TNF-α disrupts insulin signaling by enhancing c-Jun N-terminal kinase activity and simultaneously inhibiting NF-κB activity. These molecular events promote the serine phosphorylation of insulin receptor substrate-1, which disrupts normal insulin signaling and worsens insulin resistance, accelerating T2D progression (Caruso et al., 2014). This inflammatory response emphasizes IL-17 as a promising candidate for T2D management, underscoring the need for more targeted anti-inflammatory treatments. Future research should further elucidate the crosstalk between Th17 cells, insulin resistance pathways, and β-cell dysfunction to develop more effective interventions for diabetes.

The research on other IL-17 family factors and their receptors—aside from IL-17A—in diabetes is limited. IL-25 plays a vital role in diabetes by preserving gut barrier integrity by promoting tuft cell expansion and IL-25 secretion, potentially through the free fatty acid receptor 3 pathway, which helps prevent obesity and metabolic disorders (Chen et al., 2022). Additionally, IL-25 also plays a significant role in promoting diabetic wound healing. The activation of IL-17RB signaling enhances angiogenesis, collagen deposition, and endothelial function while restoring Wnt/β-catenin signaling and inducing AKT and ERK1/2 phosphorylation. IL-17RE is upregulated in diabetic nephropathy, correlating with neutrophil gelatinase-associated lipocalin, urinary albumin-to-creatinine ratio, and glomerular lesions. It serves as an independent risk factor for macroalbuminuria, exhibiting strong diagnostic value (Wang et al., 2023).

The IL-17 family impacts diabetes pathogenesis, including inflammation and insulin resistance. Targeting IL-17 signaling offers a promising strategy to improve diabetes management and outcomes.

### Inflammatory skin diseases

IL-17 has been implicated in the onset of various swelling-related skin disorders. In-depth psoriasis pathology research now recognizes IL-17A as a central effector in disease pathogenesis. Other cytokines from the IL-17 family—such as IL-17C, IL-17E, and IL-17F—are also believed to contribute to psoriasis development. Although IL-17C, IL-17E, and IL-17F are present in greater amounts than IL-17A, it is IL-17A that demonstrates the greatest biological activity (Matusiak et al., 2017). As a consequence of IL-17A signaling, keratinocytes produce proteins such as S100A7, S100A8, and S100A9, along with defensins (Noack and Miossec, 2023), leading to heightened keratinocyte growth and enhanced neutrophil recruitment, driving psoriatic plaque formation. In addition to these effects, IL-17A decreases the levels of molecules essential for late-stage differentiation, compromising epidermal barrier integrity and facilitating greater leukocyte infiltration. Among the IL-17 family members, IL-17F exhibits the greatest similarity to IL-17A, and they frequently form heterodimers. Although direct experimental data on IL-17F’s involvement in PsA is limited, indirect evidence suggests that targeting IL-17A alongside IL-17F reduces inflammation to a greater extent than targeting IL-17A alone (Glatt et al., 2018; Cole et al., 2023). IL-17C induces gene expression patterns in keratinocytes resembling those triggered by IL-17A *in vitro*, which are vital for the recruitment of immune cells (Lauffer et al., 2020). IL-17E promotes keratinocyte proliferation, differentiation (keratin 10), and motility in psoriasis, distinct from IL-17A, with no antimicrobial effects, indicating its key role in epidermal pathophysiology (Borowczyk et al., 2020).

Hidradenitis suppurativa is a skin disorder characterized by inflammation associated with Th1 and Th17 responses (Kelly et al., 2015; Rastrick et al., 2025). Histopathological examination of skin biopsy samples from hidradenitis suppurativa patients revealed an increased proportion of Th17 cells in the diseased skin (Thomi et al., 2018). Proinflammatory cytokines, including IL-17, were found at higher levels in affected skin than in the surrounding and non-affected areas. IL-17 triggers the recruitment of S100A8, S100A9, and NOD-like receptor protein 3 to the hair follicle unit and the skin surrounding the lesion (Matusiak et al., 2017). In AD, damage to the skin’s defense barrier leads to the release of IL-17E, which plays both protective and proinflammatory roles in maintaining skin balance (Spidale et al., 2020). Th17 cell counts and IL-17 concentrations were present at considerably elevated levels within the systemic circulation and scalp lesions of alopecia areata patients (Loh et al., 2018). Th17 cells influence hair follicles by releasing IL-17A, IL-21, IL-22, and IL-26, impairing hair follicle regeneration. A rise in Th17 and Th1 Inflammatory mediators (including IL-17A and IL-17F) was observed within the diseased skin of patients with pityriasis rubra pilaris. Treatment with drugs targeting IL12/23 and IL-17A—examples of which include Ustekinumab, Secukinumab, and Ixekizumab—resulted in clinical and histopathological improvement in pityriasis rubra pilaris patients (Feldmeyer et al., 2017). Increased IL-23 and IL-17 concentrations within cutaneous lesions and serum were strongly linked to disease severity in pemphigus vulgaris patients (Xue et al., 2014). In systemic sclerosis lesions, quantitative analysis demonstrated elevated IL-17A concentrations relative to those in healthy controls. Elevated IL-17A concentrations promote vascular fibrosis in systemic sclerosis patients by triggering the ERK1/2 pathway in vascular smooth muscle cells within the dermis (Seki et al., 2024).

### Systemic lupus erythematosus

Systemic lupus erythematosus (SLE) is an autoimmune disorder characterized by the loss of immune tolerance and the persistent production of autoantibodies. Research indicates that IL-17 expression levels in SLE patients differ significantly from those in healthy controls, and this differential expression is closely associated with disease initiation and progression. IL-17A is detected in both the synovial fluid and serum of SLE patients, with notably higher concentrations in the synovial fluid, predominantly secreted by CCR6^+^CD4^+^ T cells (Richter et al., 2023). This suggests a potential role of IL-17A in the pathogenesis of SLE-associated synovitis. Additionally, *IL-25* mRNA levels in peripheral blood mononuclear cells and serum are markedly elevated in SLE patients compared to healthy individuals, with higher IL-25 levels observed in patients with active disease than those with inactive disease (Li et al., 2019a). Furthermore, IL-17A, IL-17B, and IL-17F levels are significantly increased in SLE patients, and IL-17F levels exhibit a positive correlation with circulating endothelial cell counts and disease activity. IL-17F also correlates significantly with vascular endothelial growth factor and placenta growth factor, while vascular endothelial growth factor is linked to both IL-17A and IL-23 (Robak et al., 2013). These findings highlight the involvement of IL-17 family cytokines in both the inflammatory processes and potential angiogenic pathways of SLE pathophysiology.

IL-17 plays a multifaceted role in SLE. Among IL-17 family members, IL-17A is the most prominently implicated in SLE pathogenesis, while IL-17F may act synergistically with IL-17A to accelerate disease progression. Interestingly, IL-17B appears to exert a protective effect in the context of SLE. The specific roles of IL-17C, IL-17D, and IL-17E remain incompletely understood. As a key proinflammatory mediator, IL-17A is highly expressed in both synovial fluids, primarily secreted by CCR6^+^CD4^+^ T cells and serum. IL-17A promotes multi-organ damage by activating neutrophil extracellular traps (NETs). REDD1-dependent autophagy enhances the release of NETs, which carry IL-17A and tissue factor. These NETs contribute to thrombin generation and fibroblast activation, driving fibrosis, particularly in the skin lesions of discoid lupus erythematosus and renal tissue in proliferative lupus nephritis (Frangou et al., 2019). In addition, the dual role of IL-17A is reflected in the heterogeneity of its cellular sources. As well as Th17 cells, double-negative T cells secrete IL-17A via the mTORC1 signaling pathway, which is regulated by phosphatidic acid. This process promotes germinal center B cell differentiation and the production of autoantibodies (Li et al., 2024b). A subset of Tregs also secretes IL-17A, potentially contributing to immune modulation.

Notably, exosomes derived from bone marrow mesenchymal stem cells (BM-MSCs) enhance anti-inflammatory macrophage polarization and IL-17⁺Treg recruitment by downregulating PDCD4 and PTEN via miR-16 and miR-21. This regulatory mechanism plays a crucial role in mitigating lupus nephritis (Zhang et al., 2022b). IL-17F positively correlates with circulating endothelial cell counts, pro-angiogenic factors (vascular endothelial growth factor and placenta growth factor), and disease activity. By inducing the expression of *CXCL1* and *CXCL5*, IL-17F promotes neutrophil infiltration into the kidneys, exacerbating renal injury (Riedel et al., 2016). The absence of IL-17B in SLE exacerbates disease severity in lupus-prone mice, whereas exogenous IL-17B administration alleviates disease progression. Mechanistically, IL-17B suppresses aberrant B cell activation by downregulating FASN-mediated lipid metabolism, thereby inhibiting TLR and IFN signaling pathways, resulting in reduced germinal center B cell and plasma cell differentiation and ultimately mitigating the autoimmune response. Notably, SLE patients exhibit elevated *FASN* expression and reduced *IL-17RB* expression in B cells, further supporting the proinflammatory role of FASN in SLE. These findings suggest that IL-17B may serve as a novel therapeutic target, offering new insights into SLE pathogenesis (Xiao et al., 2024).

IL-25 (also known as IL-17E) is elevated in patients with active SLE and exhibits immunoregulatory functions by suppressing proinflammatory cytokines such as IL-1β, IL-6, and IL-17A. However, the precise mechanisms underlying its regulatory effects are yet to be fully elucidated (Li et al., 2019a; Selvaraja et al., 2019). The imbalance between Th17 and Tregs is a central mechanism underlying immune dysregulation in SLE. Excessive activation of Th17 cells leads to IL-17A overproduction, driving inflammation, while Treg dysfunction and autophagy activation further exacerbate this imbalance. Therapeutic strategies targeting the IL-17 pathway are becoming increasingly diverse, encompassing mechanism-based repurposing of existing drugs—such as hydroxychloroquine (inhibiting NETs formation), rapamycin (blocking the mTORC1 pathway), and triptolide (TWP, suppressing IL-17 signaling but posing hepatotoxicity risks)—as well as emerging interventions, including IL-17A/F neutralizing antibodies, IL-17B supplementation, and IL-25 agonists. However, challenges remain in achieving subtype-specific regulation, addressing cellular heterogeneity, and ensuring therapeutic safety (An et al., 2017; Xia et al., 2021).

In summary, the IL-17 cytokine family exhibits a complex role in SLE, contributing to both inflammatory responses and tissue damage, while certain subtypes may exert protective effects. Future research should further elucidate the precise mechanisms underlying these cytokines’ functions and evaluate their potential as therapeutic targets for SLE.

### Joint inflammation

The cause of joint inflammation is multifactorial and can be categorized into numerous types depending on its underlying origin. IL-17 acts as a crucial inflammatory mediator in the inflammatory processes that drive RA. Initial experiments have shown that IL-17A and IL-17F are present at highly elevated concentrations within the joint synovium of individuals with RA relative to healthy controls and osteoarthritis patients (Paradowska-Gorycka et al., 2020). IL-17A stimulates macrophages, fibroblasts, endothelial cells, and osteoblasts, triggering the secretion of proinflammatory cytokines. Importantly, TNF-α, IL-1β, IL-6, and IL-8 drive the inflammatory process in RA. These cytokines also sustain CD4^+^/IL-17^+^ T-cell populations by promoting IL-17-stimulated IL-6 production, perpetuating a vicious cycle. Furthermore, IL-17 has been demonstrated to stimulate the synthesis of chemokines, including CXCL1, CCL2, and CXCL5. These chemokines act as signals that draw monocytes and neutrophils toward the joint synovium, thereby exacerbating the inflammatory response (Chen et al., 2023; Zhang et al., 2024a). *In vitro* experiments further revealed that IL-17 stimulates bone resorption and collagen degradation. The mechanism involves IL-17 promoting the synthesis of prostaglandin E2 by osteoblasts, which subsequently induces osteoclast differentiation via the expression of osteoclast differentiation factors. Osteoclast differentiation factor binds to its receptor activator NF-κB on osteoclast progenitors, leading to osteoclast formation and causing bone resorption and collagen degradation (Kotake et al., 1999; Funaki et al., 2018). Similarly, IL-25 in RA suppresses IL-22-induced osteoclastogenesis by reducing the expression of receptor activators of NF-κB ligands. It acts through the STAT3 and p38 MAPK/NF-κB inhibitor alpha pathways, inhibits osteoclast differentiation and related marker expression, suggesting that IL-25 is a potential therapeutic target for RA (Min et al., 2020). *IL-17F* is structurally similar to IL-17A, and inhibitors targeting IL-17F, such as anakinra, have shown potential in reducing inflammation and improving disease outcomes (Nisar et al., 2023). However, specific studies detailing the exact mechanisms of IL-17F in arthritis are still lacking.

In ankylosing spondylitis (AS), the prominent feature of spinal involvement is compounded by the underlying inflammatory processes, which are central to disease progression (Daoussis et al., 2022). IL-17, a key cytokine produced during chronic inflammation, is indispensable for promoting osteoclast differentiation, resulting in increased bone loss and subsequent loss of bone density (Rosenzweig et al., 2024). Research has shown that IL-17A drives Th17 expansion and bone loss in AS by activating neutrophils. Caspase recruitment domain-containing protein 9, downstream of Dectin-1, enhances IL-17A production, with the CARD9S12N variant contributing to disease progression and bone degradation (Rosenzweig et al., 2024). IL-17A also drives inflammation and bone loss in AS by regulating the NF-κB and Th17 pathways, enhancing osteoclast activation and cytokine production, and accelerating disease progression and bone degradation (Feng et al., 2020). Notably, the production of IL-17A is stimulated by IL-17A itself, as well as by miR-214, which is upregulated in osteoblasts. This creates a feedback loop that amplifies the inflammatory response. The increased levels of IL-17A and miR-214 observed in AS patients are closely linked to disease progression, underscoring their potential as both biomarkers for diagnosis and targets for therapeutic intervention (Liu et al., 2020). Additionally, IL-17 interacts with neutrophils, accelerating the chain of events that lead to the inflammatory cascade, thereby exacerbating tissue damage. In AS, IL-17A, which is enriched in NETs, plays a key role in osteogenesis. NETs from AS patients, which carry IL-17A and IL-1β, promote MSC differentiation into bone-forming cells. IL-1β enhances *IL-17A* expression on neutrophils, and targeting IL-1β or disrupting NETs prevents osteogenesis, suggesting new therapeutic opportunities (Papagoras et al., 2021). Given the crucial role of IL-17 in the development of AS, targeting IL-17 or its associated signaling pathways represents a promising therapeutic approach. Current clinical trials indicate that upadacitinib 15 mg significantly improved the ASAS40 response in active AS patients with inadequate response to bDMARDs (45% *vs.* 18%; *P* < 0.0001), without new safety risks (van der Heijde et al., 2022). Secukinumab also significantly improved the ASAS20 (assessment of apondyloarthritis international society) response (61% and 60% at 150 mg and 75 mg), with sustained effects through 52 weeks (*P* < 0.001, *vs.* placebo) (Baeten et al., 2015). This aligns with the growing trend of precision medicine, where personalized immunomodulatory therapies can more effectively manage chronic inflammatory diseases.

In PsA, IL-17A creates an inflammatory environment that produces additional cytokines and chemokines. These molecules stimulate the recruitment of immune cells into the affected joints and activate them, further intensifying inflammation. IL-17A also stimulates target cells to produce matrix metalloproteinases (MMP-1, MMP-9, and MMP-13), thereby promoting the disintegration of the connective tissue matrix of joints. Glatt reported increased mRNA levels of *IL-17F*, along with increased levels of *IL-17A* within the synovial membrane, in PsA-affected individuals (Glatt et al., 2018). When synovial cells are subjected to TNF-α, IL-17A together with IL-17F promotes IL-8 and IL-6 synthesis. However, IL-17F was less potent than was IL-17A in this process. Suppressing the biological effects of IL-17A and IL-17F led to greater inhibition of synoviocyte and fibroblast activation than inhibiting either cytokine alone (Cole et al., 2023). Notably, IL-17A is central to the initial phase of inflammation. As inflammation progresses, the role of IL-17F becomes more prominent. These findings clarify why inhibiting IL-17A along with IL-17F results in longer-lasting disease control than does inhibiting IL-17A alone.

### Inflammatory bowel disease

Numerous studies have indicated that Th17 cells significantly contribute to the initiation of inflammatory bowel disease (IBD) (Ye et al., 2001; O’Connor et al., 2009; Jiang et al., 2014). Moreover, it should be highlighted that IL-17 is recognized as a significant contributor to this condition. In microbial infections and inflammation, when infected host cells undergo apoptosis, major histocompatibility complex class II molecules within the context of inflammation have the ability to present self-antigens (Heuberger et al., 2024). Th17 cells undergo maturation through this process, enhancing their autoreactive nature. Chemokines promote the migration of Th17 cells that are positive for the CCR6 receptor. These cells move out of the mesenteric lymph nodes to regions of intestinal inflammation. IL-17A and IL-17F aid in facilitating the expression of CCL20 in intestinal epithelial cells, thereby enhancing Th17 cell migration via the CCL20/CCR6 pathway (Hanna et al., 2022). These cells also recognize the mucosal address via cell adhesion molecule-1 on intestinal endothelial cells and venules. This recognition facilitates their migration to the intestinal mucosa via interactions between α4β7 integrin and mucosal cell adhesion molecule-1 (Uchiyama et al., 2023).

Th17 cells serve as significant contributors to IBD progression, primarily via the action of cytokines, notably IL-17A, IL-21, and TNF-α. IL-17A is particularly involved in exacerbating intestinal inflammation by inducing the upregulation of genes associated with antimicrobial peptides, inflammatory chemokines and MMPs (Marônek et al., 2021). Th17 cells and neutrophils are recruited to inflamed tissues, as IL-17A promotes IL-8 production within intestinal epithelial cells, facilitating their influx. Elevated IL-17 levels stimulate myofibroblasts to secrete MMPs, further contributing to epithelial damage and tissue remodeling (Chen et al., 2019b). IL-17A induces caspase-1-driven pyroptosis in both intestinal epithelial and stem cells within human intestinal organoids derived from adult stem cells, exacerbating intestinal injury (Lee et al., 2022). IL-17A, IL-22, and TNF-α lead to increased IL-17C secretion by colonic epithelial cells by activating the NF-κB and p38 pathways and protein-1 signaling pathways, creating an inflammatory feedback loop that sustains tissue damage (Swedik et al., 2022). However, some studies have shown that IL-17A seems to play a protective role in certain contexts of intestinal inflammation. This protective function may include immune response regulation, preservation of epithelial barrier integrity, and modulation of the gut microbiota, all of which help maintain the balance between inflammation and tissue repair.

In inflamed colonic tissues of IBD patients, the expression of *IL-17B*, in addition to that of IL-17A, has been confirmed to be upregulated (Safari et al., 2017). Single-cell analysis revealed that IL-17B deficiency exacerbates neutrophil infiltration and promotes the release of proinflammatory cytokines from intestinal macrophages. These findings suggest that IL-17B functions as an important modulator in shaping myeloid cell responses and suppressing colitis. Interestingly, neutrophil-driven inflammation, including chemotaxis, NET release, and reactive oxygen species production, may exacerbate IBD pathogenesis (Zhang et al., 2023). Moreover, the imbalance between the gut microbiota and immune homeostasis is closely associated with IBD. IL-17C in IBD is induced by the presence of gram-negative bacteria and epithelial activation. Rare loss-of-function variants in dual NADPH oxidase 2 are linked to increased IL-17C levels, which are associated with the mucosal expansion of Proteobacteria. IL-17C serves as a marker for disrupted microbiota-immune homeostasis, contributing to IBD pathogenesis (Grasberger et al., 2021).

On the basis of findings from basic research, targeting the IL-17 signaling pathway has potential as a novel therapeutic strategy for IBD. Nonetheless, clinical trials have demonstrated that secukinumab, an IL-17A inhibitor that is effective in treating psoriasis and PsA, is associated with the onset or exacerbation of IBD, including Crohn’s disease and ulcerative colitis. Retrospective studies present mixed results, with some patients—particularly those with a history of IBD or family history—experiencing new-onset or flare-up of IBD. Appropriate screening and risk stratification are recommended to minimize susceptibility to secukinumab-induced IBD (Ali et al., 2021).

### Cancer

IL-17 and its family members play pivotal roles in shaping the tumor immune microenvironment (TME), influencing tumor progression, immune evasion, and therapeutic responses (Su et al., 2010; Keerthivasan et al., 2014; Qian et al., 2017). These cytokines interact with immune cells, stromal components, and key signaling pathways, which can either promote or suppress tumor growth, depending on the context.

In pancreatic ductal adenocarcinoma, IL-17A promotes tumor progression by inducing the differentiation of cancer-associated fibroblasts via IL-17RA and TNF signaling, creating a tumor-promoting stromal environment. Tc17 cells, which produce IL-17A, increase tumor cell proliferation and survival, contributing to poor prognosis (Picard et al., 2023). Similarly, in colorectal cancer, IL-17A increases resistance to anti-PD-1 therapy by upregulating *PD-L1* (programmed death-ligand 1) expression through the P65/nuclear respiratory factor 1/miR-15b-5p axis (Liu et al., 2021). IL-17A reshapes the TME by mobilizing myeloid-derived suppressor cells and suppressing cytotoxic T lymphocytes, ultimately impairing antitumor immunity and therapeutic efficacy (Jou et al., 2022). These findings highlight IL-17A as a critical player in immune evasion and therapeutic resistance across various cancers. IL-17A and related cytokines also play significant roles in modulating immune cells within the TME. In lung cancer, IL-17D enhances tumor progression by recruiting macrophages to the tumor site and activating the p38 MAPK signaling pathway, promoting macrophage polarization. This process contributes to immune evasion, as tumor-associated macrophages are associated with increased tumor growth and poor prognosis (Lin et al., 2022b). Similarly, IL-17A and IL-17F contribute to immune cell recruitment and polarization. In lung cancer, IL-17A/F promotes the adoption of an M2-like phenotype by macrophages, which enhances tumor cell migration, proliferation, and angiogenesis, thereby supporting tumor progression (Ferreira et al., 2020). In colon cancer, IL-25-activated ILC2s create a permissive microenvironment for tumor growth by recruiting myeloid-derived suppressor cells and impairing antitumor immunity. These interactions demonstrate how IL-17 family cytokines orchestrate immune cell behavior, playing a central role in shaping the TME to favor tumor growth. Notably, IL-17A has tumor-suppressive effects under certain conditions. Compared with wild-type mice, IL-17A-deficient mice presented larger melanoma tumor volumes and increased lesion burdens (Chen et al., 2019b). Conversely, adoptive T-cell therapy utilizing tumor-specific Th17 cells was shown to activate tumor-specific CD8^+^ T cells, thereby suppressing tumor progression (Xiao et al., 2023). Furthermore, a study revealed that IL-17A induces mitochondrial dysfunction in colorectal cancer cells by activating the ROS/NOD-like receptor protein 3/caspase-4/gasdermin D pathway, stimulating intracellular ROS production and promoting tumor cell pyroptosis (Feng et al., 2023). Additionally, IL-17F has been demonstrated to inhibit the proliferation and migration of oral tongue squamous cell carcinoma cell lines (SCC-25 and HSC-3) and to significantly suppress angiogenesis (Almahmoudi et al., 2021).

In addition to immune modulation, IL-17A is involved in tumor-induced bone resorption. In lung cancer, IL-17A promotes osteoclastogenesis by regulating osteoclast precursor apoptosis, thus enhancing osteoclast survival and contributing to bone destruction. IL-17A deficiency impairs osteoclast differentiation and osteolytic activity, indicating that targeting IL-17A could be a promising therapeutic strategy for treating cancer-related bone metastasis (Wang et al., 2024). Furthermore, in colon cancer, IL-17F induces pyroptosis in endothelial cells through the Caspase 4/gasdermin D signaling pathway. This process leads to the expression of the gasdermin D N-terminus, cellular damage, and the suppression of angiogenesis. IL-17F also modulates the TME by recruiting CCR6^+^ immune cells, underscoring its potential as a therapeutic target to inhibit tumor vasculature and progression (Zhou et al., 2024). IL-17 also contributes to immune escape by activating immune-suppressive pathways. In lung cancer, the protein BTNL2 further suppresses antitumor immunity by enhancing IL-17A production and recruiting myeloid-derived suppressor cells. Inhibiting BTNL2 reduces the number of IL-17A^+^ γδ T cells, increases the presence of cytotoxic CD8^+^ T cells, and slows tumor progression (Du et al., 2022). Similarly, IL-17D in lung cancer facilitates immune evasion by enhancing macrophage recruitment to the tumor site via the p38 MAPK signaling pathway. IL-17D overexpression increases macrophage polarization and infiltration, promoting tumor growth and progression. Targeting IL-17D may offer a valuable strategy to counter immune escape and improve therapeutic outcomes in patients with lung cancer (Lin et al., 2022b). Moreover, Act1, a key component of the IL-17R complex, has dual functions in signaling and controlling mRNA stability, further influencing gene expression and inflammatory responses (Chen et al., 2022).

Recent clinical research has indicated that high IL-17A levels in early-stage colorectal cancer (stage I or II) correlate strongly with rapid tumor progression and metastasis, suggesting that IL-17A could serve as a reliable prognostic biomarker (Fakih et al., 2023). Moreover, early-stage evidence suggests that combining IL-17A inhibition with immune checkpoint inhibitors can increase therapeutic effectiveness by mitigating the immunosuppressive effects of the TME. This approach holds promise for augmenting anticancer therapy outcomes across several malignancies, including non-small cell lung cancer, pancreatic cancer, and gastric cancer (Nagaoka et al., 2020; Zhang et al., 2020; Shi et al., 2023). These findings indicate that IL-17A and its signaling pathways could represent crucial therapeutic targets for enhancing antitumor immunity in solid tumors.

In conclusion, IL-17 cytokines play diverse and complex roles in tumor progression and immune modulation, from promoting immune evasion to enhancing tumor angiogenesis and metastasis. These findings underscore the ability of the IL-17 family of cytokines to serve as therapeutic targets in cancer treatment, offering new avenues for improving the immune response and overcoming treatment resistance. By targeting IL-17 signaling, enhancing antitumor immunity, reducing immune suppression, and improving the clinical outcomes of cancer patients may be possible.

## Therapeutic Approaches Targeting Interleukin-17/T helper 17

There are increasing reports of autoimmune diseases and IL-17/Th17. These diseases have been the second-largest market for immunotherapy, with significant unmet clinical needs. Autoimmune inflammation is characterized by dysregulated immune cell activation, inflammatory cytokines secretion, and altered intracellular signaling. While each autoimmune disease has a unique pathophysiology, key molecules in common inflammatory pathways can serve as immunotherapeutic targets. Following the success of PD-1, many domestic antibodies targeting autoimmune pathways have advanced rapidly, with IL-17/IL-17R emerging as a competitive focus (Li et al., 2021b). Interest in IL-17/IL-17R antibodies is driven by the significant role IL-17 plays in diverse biological processes. IL-17 ligands bind their receptors to induce organ-specific inflammatory responses. IL-17RA, the initial receptor identified within the IL-17R family, plays a crucial role as a co-receptor for multiple ligands belonging to the IL-17 cytokine group, facilitating their binding and initiating downstream signaling pathways. Currently, five biologic therapies targeting IL-17/IL-17R are available globally, comprising four monoclonal antibodies against IL-17 (Novartis’ Secukinumab, Ixekizumab, Biocad’s Netakimab, and UCB’s Bimekizumab) and one monoclonal antibody targeting IL-17RA (UCB’s Brodalumab) (He et al., 2021). Numerous clinical research drugs are in development. The future of IL-17-targeted therapies is shifting toward long-term and oral administration, which is more patient-friendly. DICE Therapeutics (South San Francisco, CA, USA), for instance, developed two oral IL-17 small-molecule antagonists, DC-806 and DC-853, based on the DELSCAPE technology platform, known for its efficacy and innovative design. On June 20, 2023, Lilly (Indianapolis, IN, USA) acquired DICE for USD 2.4 billion (**Table 3**; Berger et al., 2024).

Secukinumab, the inaugural antibody directed against IL-17A, was authorized in 2015 for treating plaque psoriasis, PsA, and AS (Baeten et al., 2015; Mease et al., 2015; Rothstein and Gottlieb, 2016). Subsequently, Ixekizumab and Brodalumab were also authorized for the management of psoriasis and other conditions linked to IL-17 or IL-17R signaling (Lebwohl et al., 2015; Gordon et al., 2016; Armstrong and Read, 2020; Yiu et al., 2022). Secukinumab achieved significant psoriasis area and severity index (PASI) 100 responses (48.9% at week 16, 46.2% at week 48) and rapid improvements (47.3% with 75% PASI reduction at week 4). It demonstrated a higher American College of Rheumatology 20 (ACR20) response in patients with concomitant PsA (76.4%) (Gottlieb et al., 2022). Common side effects include oral candidiasis (3.0%).

While less effective than Bimekizumab in terms of PASI 100 responses, Secukinumab remains a strong treatment option with acceptable safety and efficacy profiles (Gottlieb et al., 2022). A multicenter study found no significant association between genetic variants in the IL-17A gene and treatment responses to Secukinumab or Ixekizumab. Both treatments exhibited significant efficacy, with high PASI75 and PASI90 response rates at 12 and 24 weeks (van Vugt et al., 2020). However, due to limited or inadequate clinical outcomes, several studies evaluating Secukinumab and Brodalumab in other IL-17-associated conditions were terminated (Ben-Anaya et al., 2024). Previous research demonstrated that anti-IL-17 monoclonal antibody therapy failed to prevent relapse in a relapsing-remitting EAE model. Recent research has shed light on the molecular mechanisms involved, demonstrating that IL-17 activates SH2 domain-containing protein-tyrosine phosphatase-2 and autonomously sustains IL-17R signaling even when IL-17 is absent. This finding enhances our understanding of this well-characterized pathway, offering new insights for therapeutic use. The study also suggests that similar mechanisms of receptor signal autonomy might apply to other autoimmune diseases, offering insights into alternative inflammatory signaling pathways and opening new avenues for the remediation of chronic diseases (Luo et al., 2023).

The functional synergy among IL-17 family cytokines provides a unique rationale for developing novel therapeutic agents. Bimekizumab, as the first humanized monoclonal antibody simultaneously targeting IL-17A and IL-17F, overcomes the limitations of conventional single-cytokine targeting through its dual inhibitory mechanism. Its design is based on the substantial overlap between IL-17A and IL-17F in their structural homology, receptor sharing, and activation of downstream signaling pathways (Reich et al., 2021). A preclinical study has demonstrated that IL-17A and IL-17F synergize with TNF to stimulate the production of pivotal proinflammatory cytokines and amplify tissue inflammation (Dixon et al., 2022). Compared with isolated IL-17A blockade, dual neutralization of IL-17A/F induces more profound suppression of inflammation-associated gene/cytokine expression and exerts greater inhibitory effects on the migration of disease-relevant immune cells (Reich et al., 2021). By binding conserved epitopes shared by both IL-17A and IL-17F, Bimekizumab effectively blocks their receptor interactions, thereby achieving comprehensive inhibition of Th17 cell-mediated inflammatory cascades (Freitas et al., 2021).

In clinical trials, all Bimekizumab dosage groups demonstrated superior efficacy over placebo across primary and secondary endpoints in moderate-to-severe psoriasis treatment. Notably, approximately 50%–60% of patients in the three highest dose cohorts achieved PASI 100 by week 12. Compared with therapies targeting IL-17A, IL-17RA, or IL-23 alone, this trial suggested potentially faster achievement of high-level skin clearance (Ruggiero et al., 2022; Zozaya et al., 2022). The safety profile of Bimekizumab aligns with that of other IL-17 inhibitors, with common adverse events, including mild upper respiratory infections and injection-site reactions, and without significant elevation of candidiasis risk. Currently approved in the European Union and USA for psoriasis and PsA, Bimekizumab is undergoing phase III trials for Crohn’s disease and hidradenitis suppurativa (Kokolakis et al., 2023; Kimball et al., 2024). Future investigations should elucidate tissue-specific variations in IL-17A/F synergy to optimize targeted therapeutic strategies.

Brodalumab, a fully human monoclonal antibody against IL-17RA, exemplifies a receptor-level strategy to disrupt the functional synergy of IL-17 family cytokines. By binding to IL-17RA—the shared receptor subunit for IL-17A, IL-17F, IL-17C, and IL-17E (IL-25)—Brodalumab broadly inhibits signaling across multiple IL-17 heterodimeric complexes (Issa and Kircik, 2023). This pan-IL-17 blockade addresses the compensatory upregulation of alternative IL-17 isoforms observed in single-cytokine-targeted therapies. Mechanistically, IL-17RA is essential for the assembly of functional receptor complexes and the activation of downstream proinflammatory mediators. Preclinical evidence indicates that concurrent inhibition of IL-17A and IL-17F via IL-17RA blockade synergistically reduces keratinocyte hyperproliferation and neutrophil chemotaxis in psoriatic models, achieving superior suppression of IL-23/Th17 axis-driven inflammation compared to IL-17A-specific neutralization (Glatt et al., 2018). In clinical trials, Brodalumab-treated subjects exhibited a higher incidence of Candida infections than the placebo group, although severe infections remained uncommon (Bilal et al., 2024). Early-phase trials reported suicidal ideation in 0.3% of participants, prompting the US Food and Drug Administration (FDA) to issue a boxed warning; however, subsequent analyses failed to establish a causal relationship (Chiricozzi et al., 2016). Currently approved in multiple countries for psoriasis and PsA treatment, Brodalumab’s receptor-centric targeting strategy demonstrates potential therapeutic value for diseases involving complex interactions among IL-17 subtypes. Current investigations are focusing on elucidating tissue-specific IL-17R dynamics to refine its therapeutic window.

Given the suboptimal clinical outcomes of IL-17-targeted therapies in neuroimmunological diseases, some studies have shifted toward targeting IL-17-producing cells. IL-17A and IL-17F are primarily derived from Th17 cells, an important pathogenic T cell subset critical for the manifestation of these conditions. By mediating immune responses, Th17 cells play a crucial role in initiating and advancing autoimmune disorders. They are essential for neutrophil recruitment (Warnatsch et al., 2015; Stone et al., 2024), inflammation (Damasceno et al., 2020), and host defense (Zhu et al., 2024). However, when dysregulated, they can lead to tissue damage. CD20 monoclonal antibodies help regulate inflammation by depleting Th17 cells directly and reducing B cell activation indirectly (Du et al., 2017). CD20-targeted therapy has improved outcomes in RA by suppressing Th17 responses (Gupta et al., 2023). Similarly, in a right middle occipital gyrus-induced mouse model, CD20 antibodies reduced both Th1 and Th17 subsets in the CNS, demonstrating its potential in managing autoimmune-related immune activity (Weber et al., 2010; Holloman et al., 2021). In an EAE model, myelin oligodendrocyte glycoprotein-specific B cells worsened Th17-driven disease, and B cell inhibition indirectly hindered Th17 differentiation (Fazazi et al., 2024).

Anti-CD20 therapy in MS targets B cells, particularly those not actively producing antibodies, to reduce their numbers and modulate the immune response. This approach relies on several mechanisms, such as antibody-dependent cellular cytotoxicity and complement-dependent cytotoxicity, all of which work synergistically to promote CD20^+^ B cell eradication. These B cells are implicated in the development of MS, contributing to CNS inflammation and demyelination. The primary anti-CD20 drugs used in MS treatment include Rituximab, Ofatumumab, Obinutuzumab, and Ocrelizumab, all of which have demonstrated significant efficacy in reducing disease relapse and progression by targeting B cells (Florou et al., 2020). In a cohort study, Rituximab showed a higher annualized relapse rate than Ocrelizumab (annualized relapse ratio 1.8, *P* < 0.001), although no difference in disability progression was observed (Roos et al., 2023). Ocrelizumab demonstrated high efficacy, significantly reducing relapse rates and disease activity in relapsing MS and primary progressive MS, with effects maintained over 7.5 years (Lamb, 2022). It is well tolerated, with no new safety signals and involving biannual infusions. Ofatumumab demonstrated a low annualized relapse rate (0.05) and fewer lesions, meeting the “no evidence of disease activity” criteria in 78.8% of patients over 4 years. Infections were the most common side effect (Hauser et al., 2023). Obinutuzumab shares similar mechanisms to Rituximab and Ocrelizumab but the literature lacks specific data on clinical efficacy and safety. Overall, Rituximab and Ocrelizumab are effective, with Ocrelizumab preferred for primary progressive MS, while Ofatumumab showed promising efficacy with a good safety profile (Lamb, 2022).

CD19 is another important cell surface marker expressed on B cells that regulates their activation, proliferation, and interaction with T cells. One promising therapy targeting CD19 is Inebilizumab, an IgG1 monoclonal antibody developed by Viela Bio (Gaithersburg, MD, USA). Inebilizumab enhances antibody-dependent cellular cytotoxicity, leading to the efficient depletion of B cells and plasma cells (Chen et al., 2016). Granted approval by the FDA in June 2020, Inebilizumab became the second FDA-endorsed therapy to treat NMOSD, an uncommon yet severe autoimmune disorder affecting the CNS. The NMO-mentum study (NCT02200770) showed that Inebilizumab effectively reduced NMOSD attacks in AQP4-IgG-seropositive patients, with an annualized attack rate of 0.052 attacks/person-year. Disability scores remained stable over ≥ 4 years. It was well tolerated, with minimal serious AEs (SAEs) (2.7%) and no deaths (Cree et al., 2021). The phase III MITIGATE trial (NCT04540497) showed that Inebilizumab significantly reduced IgG4-related disease flares, with 10% of patients in the treatment group experiencing flares *vs*. 60% in the placebo group (Hazard ratio 0.13, *P* < 0.001). It also improved flare-free complete remission rates. SAEs occurred in 18% of Inebilizumab-treated patients (Stone et al., 2025). In a phase I trial (NCT02200770), Inebilizumab (MEDI-551) exhibited dose-dependent B cell depletion and improved skin thickness in systemic sclerosis patients, with a mean change of –5.4 ± 4.2 in modified Rodnan skin score. Side effects, including nausea and fatigue, were common; pulmonary function was unaffected (Campochiaro and Allanore, 2021). These trials further underscore the growing role of B cell depletion therapies for managing autoimmune diseases.

Several indirect IL-17-targeting drugs have also been marketed, including inhibitors of IL-6/IL-6R, IL-1, and IL-23, that are key in controlling Th17 differentiation. Small molecules that target RORc can help restore the equilibrium between Th17 and Treg cell populations. Additionally, administering IL-2 can further support the restoration of this equilibrium. What is the current application of these indirect IL-17 inhibitors in neuroimmunological diseases? On August 15, 2020, the FDA officially endorsed Roche’s IL-6R antibody, Satralizumab, to treat NMOSD in pediatric and adult patients, making it the third medication to receive approval for this condition. In the phase III trial (NCT02073279), Satralizumab reduced NMOSD relapse rates compared to placebo (Hazard ratio 0.45, *P* = 0.018). AEs, including headaches and upper respiratory infections, were similar between groups, with no significant difference in SAEs, supporting its favorable safety profile (Traboulsee et al., 2020). Our team discovered that interleukin-1 receptor-associated kinase M in microglial cells promotes inflammasome-mediated IL-1β production, which in turn promotes microglial survival through a feedback mechanism and exacerbates myelin damage by enhancing Th17 cell inflammatory functions. This new mechanism suggests that blocking IL-1β or IL-1R signaling within the CNS system may serve as an innovative approach to preventing myelin damage. Novartis’ IL-1β inhibitor ILARIS® (Canakinumab) is FDA-approved for periodic fever syndromes and Still’s disease, but no clinical trials have yet been conducted for neuroimmunological diseases.

IL-23-targeting biologics, including Ustekinumab, Guselkumab, Tildrakizumab, and Risankizumab, are mainly prescribed for managing psoriasis, a long-lasting autoimmune condition characterized by skin inflammation and irregular cell proliferation. Ustekinumab, the first biologic to target IL-23, also inhibits IL-12, another cytokine involved in immune regulation. By targeting both IL-12 and IL-23, Ustekinumab acts as a broader immunomodulator rather than as a specific IL-23 inhibitor. Despite its effectiveness in managing moderate-to-severe psoriasis, Ustekinumab’s broader action may limit its specificity in targeting the precise cytokine pathways involved in skin inflammation. In contrast, Guselkumab, approved in July 2017, was the first biologic specifically targeting IL-23, making it a more targeted and selective therapy for psoriasis. By 2020, Guselkumab had received approval from the FDA and the European Medicines Agency, reinforcing its role as a key treatment option. Research has shown that Guselkumab and Risankizumab (approved in 2019) offer superior efficacy in comparison to Ustekinumab, highlighting IL-23 as a pivotal factor in psoriasis development. Meanwhile, Tildrakizumab, another selective IL-23 inhibitor, was approved in 2018 and is also being explored for treating PsA. Despite their successes in psoriasis, IL-23 inhibitors have demonstrated modest effectiveness in treating axial spondyloarthritis, highlighting the complexity of immune modulation in different autoimmune conditions.

A phase II clinical study of a new anti-IL-23p19 monoclonal antibody was conducted in the given context, with 75% of individuals suffering from moderate-to-severe plaque psoriasis achieving a PASI 75 by week 16, demonstrating promising efficacy for IL-23 inhibition in psoriasis treatment. Ongoing phase III trials for Mirikizumab, an anti-IL-23p19 monoclonal antibody, are registered under NCT03482011, NCT03535194, and NCT03556202, and are expected to further confirm its clinical potential. Among the IL-23 inhibitors, Tildrakizumab has demonstrated lower efficacy compared to others like Guselkumab and Risankizumab, which may be due to its weaker binding affinity for the p19 subunit of IL-23. Specifically, Guselkumab has a dissociation constant (Kd) of 3.3 pM, indicating stronger binding, while Tildrakizumab’s Kd is considerably higher at 297 pM (Puig, 2019; Santostefano et al., 2019). In clinical practice, both Guselkumab and Risankizumab have demonstrated higher response rates in treating psoriasis than TNF inhibitors, offering more effective options for patients and elucidating the key role of IL-23 in psoriasis pathogenesis.

Furthermore, direct comparison trials between Risankizumab and Ustekinumab demonstrated that Risankizumab-treated patients achieved a PASI 90 score by week 16 at a higher rate. Patients who do not respond adequately to Ustekinumab may improve by transitioning to a selective IL-23 inhibitor. Research has shown that IL-23 inhibitors, such as Risankizumab and Guselkumab, are more effective than Ustekinumab, reinforcing the crucial involvement of IL-23 signaling in psoriasis pathogenesis. IL-23 stimulates the production of IFN-γ-secreting Th1 cells, which in turn inhibit Th17 cell development. Additionally, preclinical data suggest that IL-12 may reduce inflammation in keratinocytes, and the neutralization of IL-23 has been shown to improve symptoms of PsA.

**Figure 6:**
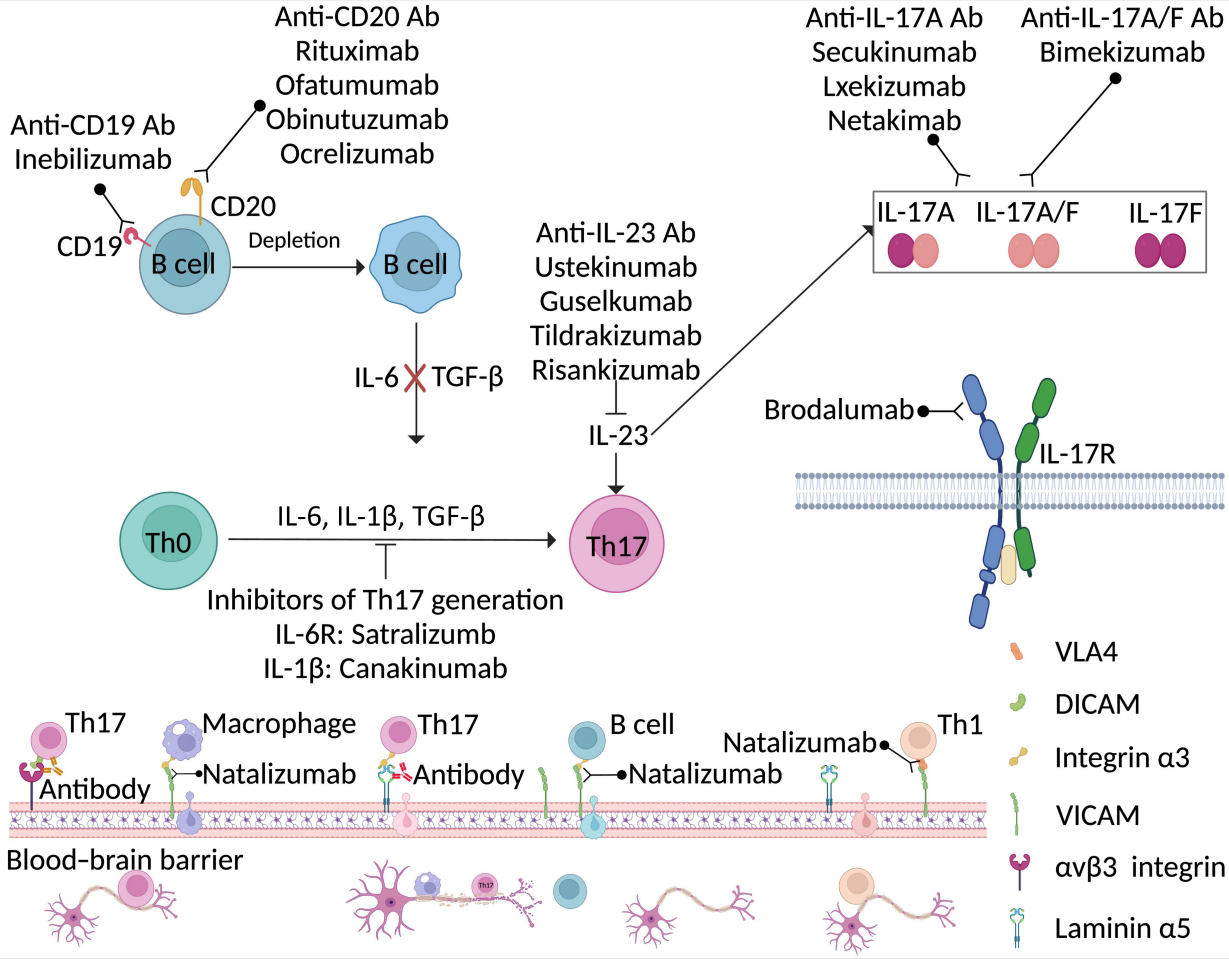
Blocking mechanisms of interleukin-17 signaling in immune diseases. The addition of IL-6 + TGF-β or IL-6 + IL-1β + IL-23 to naïve mouse CD4^+^ T cells is required for T cells to upregulate RORγt and produce Th17 cytokines. IL-17 signaling can be achieved by blocking interleukin-17A (Secukinumab, Lxekizumab, and Nanakimab) and interleukin-17F (Bimekizumab) antibodies. IL-6 and TGF-β secreted by B cells are important inducers of Th17 cells. Antibodies targeting CD20 (Rituximab, Ofatumumab, Obinutuzumab, or Ocrelizumab) and CD19 (Inebilizumab) can lead to B-cell exhaustion and indirectly hinder the differentiation of Th17 cells. Inhibitors of IL-6R (Satralizumab), IL-1β (Canakinumab) or IL-23 (Uselkumab, Tildrakizumab, and Risankizumab), which indirectly target the IL-17 signaling pathway, can also affect Th17 cell differentiation. Blocking the infiltration of immune cells into the CNS can prevent autoimmune neuroinflammation. Natalizumab is an approved monoclonal antibody designed to specifically hinder this process by targeting VLA4/VCAM1, an integrin expressed on almost all immune cell subsets. DICAM and Integrin α3 are characteristically expressed on Th17 cells and bind to αvβ3 integrin and Laminin α5 on the blood‒brain barrier, respectively. Anti-DICAM and anti-αvβ3 integrin antibodies reduced the number of Th17 cells in the CNS. Created with BioRender.com. CD19: Cluster of differentiation 19; CD20: Cluster of differentiation 20; DICAM: dual immunoglobulin domain containing cell adhesion molecule; IL-1β: interleukin-1β; IL-6: interleukin-6; IL-17: interleukin-17; IL-17A: interleukin-17A; IL-17F: interleukin-17F; IL-17R: interleukin-17 receptor; IL-23: interleukin-23; TGF-β: tumor growth factor-β; Th0: helper T-cell 0; Th1: helper T-cell 1; Th17: helper T-cell 17; VICAM: vascular cell adhesion molecule 1; VLA4: very late antigen 4.

Tildrakizumab and Risankizumab are being developed as potential therapies for PsA, while Guselkumab received FDA and European Medicines Agency approval in 2020. Interestingly, IL-23 inhibition has proven ineffective in axial spondyloarthritis. Patients with prominent psoriatic skin lesions but no axial involvement may benefit most from IL-23 inhibition. Furthermore, IL-23 inhibitors are an ideal choice for patients with lower treatment adherence or those needing longer injection intervals. In specific cases, such as in patients with a history of cancer or with a tendency for Candida infections, antibodies targeting IL-23 might be a preferred choice over biologics targeting IL-17 or TNF (**Figure 6**).

Using treatment/intervention keywords of “Secukinumab,” “Ixekizumab,” “Netakimab,” “Brodalumab,” and “Bimekizumab” and selecting only trials with reported results, we identified a total of 198 eligible trials from a search of ClinicalTrials.gov and the Chinese Clinical Trial Registry. These trials primarily focused on psoriasis (43%), AS (26%), PsA (16%), and RA (6%). Most of the studies were in phase II or III, assessing the drug’s efficacy and safety.

Drugs such as Secukinumab and Ixekizumab are currently clinically applied in various immune-mediated diseases. In a multicenter, randomized controlled clinical trial (NCT02159053), Secukinumab was used to treat AS to evaluate its efficacy over 16 weeks of treatment and its safety and effectiveness over 2 years. After 16 weeks of treatment, the ASAS20 responses for the Secukinumab 150 mg with loading dose and Secukinumab 150 mg without loading dose groups (hereinafter referred to as the With and Without groups) were 60.5% and 65.5%, respectively—11.4% and 16.4% higher than the placebo group—while the ASAS40 responses reached 10% and 8.7%, respectively. Further measurement of serum C-reactive protein levels showed a decrease of 4.23 and 6.57 in the With and Without groups, respectively, while the placebo group increased by 0.62. Numerous outcome measures indicated the significant efficacy of Secukinumab in treating AS. The trial also followed AEs and SAEs in participants over the 104-week study period. The mortality rate was 1.72% among the 116 participants in the With group, whereas the other groups had no mortality. SAEs, such as anemia, osteoarthritis, and uterine leiomyomas, occurred in 15.52% and 9.40% of the With and Without groups, respectively, compared to 3.42% in the placebo group. In terms of general AEs, such as diarrhea, cough, and hypertension, the rates were 77.59%, 76.07%, and 46.15%, respectively. Although the trial results indicated that the majority of Secukinumab-related side effects were mild, the clinical use of IL-17 inhibitors requires close monitoring, particularly during long-term treatment, to ensure patient immune tolerance and safety.

Another clinical trial analyzed the effects of Ixekizumab in Chinese patients with moderate-to-severe plaque psoriasis (NCT03364309). This study included 438 participants categorized into three groups according to the dosing regimen: Ixekizumab 80 mg Q4W (Q4W group), Ixekizumab 80 mg Q2W (Q2W group), and a placebo group. The results were evaluated after 12 weeks of treatment, finding that the Q4W and Q2W groups achieved PASI75, PASI90, and PASI100 responses of 87.4%, 75.9%, 29.3% and 93.8%, 82.4%, 33.0%, respectively, whereas the placebo group achieved only 8.0%, 2.3%, and 0%. The percentage of patients achieving sPGA score clearances was 35.6% and 36.4% in the Q4W and Q2W groups, respectively, compared to 0% in the placebo group. A range of assessment criteria further supported the efficacy of Ixekizumab in treating plaque psoriasis. The safety of Ixekizumab was analyzed during a 72-week follow-up period. Notably, there were no deaths in any of the groups, and surprisingly, the incidence of SAEs was higher in the placebo group compared to the Q4W and Q2W groups. The rate of general AEs in the Q4W and Q2W groups was 51.15% and 55.11%, substantially greater than the placebo group (28.41%). A study on Brodalumab’s efficacy and safety in patients with PsA (NCT02024646) showed that, after 16 weeks of subcutaneous injection of 210 and 140 mg, the ACR20 was 17.1% and 24.2% higher compared to the placebo group, while the AE rates were similar across all three groups. In summary, a large number of trial cases have demonstrated the significant therapeutic effects of Secukinumab and other drugs in various immune diseases, although these treatments also carry a range of side effects and potential issues related to resistance.

Despite the significant success of IL-17 inhibitors in treating many immune diseases, there are still challenges in clinical application. Drugs such as Ixekizumab, Secukinumab, and Bimekizumab may elevate susceptibility to upper respiratory infections or nasopharyngitis in the short term, with Bimekizumab showing a notable association with mucosal fungal infections in areas such as the mouth and esophagus. Some patients treated with IL-17 inhibitors may experience exacerbation of IBD, such as Crohn’s disease and ulcerative colitis, with Brodalumab being contraindicated in patients with Crohn’s disease. In a Secukinumab trial for treating severe Crohn’s disease (NCT01009281), the treatment did not significantly reduce Crohn’s Disease Activity Index ≥ 50 compared to placebo, and the study was prematurely terminated due to a higher incidence of fungal infections in the Secukinumab group. Resistance is another significant challenge. Prolonged use of these biologics can lead to the development of anti-drug antibodies (ADAs), which may neutralize the drug’s efficacy. The presence of ADAs may shorten the drug’s half-life in the body and reduce its effectiveness. In a study of Ixekizumab for moderate-to-severe psoriasis with 1346 participants (NCT01646177), 17.4% of patients developed treatment-related anti-drug antibodies (TE-ADAs) over 60 weeks, and nine patients developed high-titer TE-ADAs, demonstrating a poor clinical response (Reich et al., 2018). Similarly, in two other studies on Ixekizumab for moderate-to-severe psoriasis (NCT01474512 and NCT01597245), 42 of 220 patients (19.1%) developed ADAs, and 39 patients developed ADAs after treatment cessation and subsequent relapse (Blauvelt et al., 2017). Additionally, 19%/39% of psoriasis patients tested positive for ADAs after 20/36 weeks of Bimekizumab treatment (NCT02529956 and NCT03025542) (Glatt et al., 2017; Oliver et al., 2022).

It is unwise, however, to take an overly negative view of these side effects. Bimekizumab treatment for moderate-to-severe psoriasis was linked to a low rate of serious infections (1.0/100 person-years), and despite psoriasis patients having a higher risk for IBD, the incidence of IBD during Bimekizumab treatment was only 0.1/100 person-years. Although IL-17 and IL-23 inhibitors may increase the risk of some infections over the short term, long-term use has not been linked to a higher likelihood of severe infections or cancers. Furthermore, the risk of herpes zoster infection in psoriasis patients treated with IL-17 inhibitors does not significantly increase, with most reports indicating that the incidence of herpes zoster is lower or almost absent.

Although IL-17 plays a crucial role in neutrophil recruitment and activation, the frequency of neutropenia induced by Secukinumab in psoriasis, PsA, and AS is low, at 0.3, 0.2, and 0.5/100 person-years, respectively. Given the global coronavirus disease 2019 (COVID-19) pandemic, the relationship between IL-17 inhibitors and COVID-19 infection has raised significant concerns. Research by Liu et al. (2022) and Schmidt (2021) indicates that IL-17 inhibitors do not elevate the risk of severe acute respiratory syndrome coronavirus 2 (SARS-CoV-2) infection or exacerbate the progression of COVID-19 in psoriasis patients. Compared to Ixekizumab, Secukinumab and Brodalumab are fully human antibodies, thus have lower immunogenicity (Hsu and Armstrong, 2013). In a study on Secukinumab for severe plaque psoriasis (NCT01009281), the incidence of TE-ADA was consistently below 1%, even with treatment durations of up to 268 weeks, indicating Secukinumab’s low immunogenicity. Among the few TE-ADA-positive cases, half were temporary, with low antibody titers, and there was no clear correlation with Secukinumab dosage or treatment regimen. Most TE-ADA-positive patients showed normal pharmacokinetic profiles when using a fixed 4-week dosing interval without experiencing efficacy loss or TE-ADA-related side effects (Hsu and Armstrong, 2013).

Several studies on Brodalumab in psoriasis have shown low or no occurrence of ADAs, and even when ADAs were detected, neutralizing antibodies were absent, and clinical efficacy remained unaffected. One study (EudraCT No. 2018-000097-30) treated 20 psoriasis patients with 210 mg Brodalumab weekly for 3 weeks, followed by bi-weekly dosing. After 12 weeks, no ADAs were detected in any sample. Even in studies where ADAs were present, there was no significant reduction in clinical response (Papp et al., 2014; Bagel et al., 2020; Enevold et al., 2022). In summary, the differential response to IL-17 inhibitors among patients may be influenced by genetic background, immune system status, and other factors, which makes predicting treatment efficacy a challenge (**Table 4**).

**Table 4:** Clinical trial outcomes of partial inhibitors in autoimmune diseases

Trial number	Disease type	Drug name	Trial phase	Primary endpoint	Summary of Results
NCT02159053	Ankylosing spondylitis	Secukinumab	Phase III	ASAS 20 (16 wk)	ASAS 20 response rates: Secukinumab 150 mg with loading dose group (60.5%) Secukinumab 150 mg without loading dose group (65.5%) Placebo group (49.1%)
NCT03364309	Plaque psoriasis	Ixekizumab	Phase III	sPGA (12 wk) PASI 75 (12 wk)	sPGA responder rate: Ixekizumab 80 mg Q4W (79.9%) Ixekizumab 80 mg Q2W (86.4%) Placebo group (3.4%) PASI 75 rate: Ixekizumab 80 mg Q4W (87.4%) Ixekizumab 80 mg Q2W (93.8%) Placebo group (8.0%)
NCT02024646	Psoriatic arthritis	Brodalumab	Phase III	ACR20 (16 wk)	ACR 20 response rate: 210 mg Brodalumab (41.8%) 140 mg Brodalumab (48.9%) Placebo group (24.7%)
NCT01009281	Crohn’s disease	Secukinumab	Phase II	–	The clinical trial was prematurely terminated due to insufficient therapeutic efficacy and an elevated incidence of adverse evens. Percentage of participants with adverse evens (42.9%)
NCT01646177	Psoriasis	Etanercept Ixekizumab	Phase III	sPGA (12 wk) PASI 75 (12 wk)	sPGA responder rate: Ixekizumab 80 mg Q4W (75.4%) Ixekizumab 80 mg Q2W (80.5%) 50 mg Etanercept (41.6%) Placebo group (6.7%) PASI 75 rate: Ixekizumab 80 mg Q4W (84.2%) Ixekizumab 80 mg Q2W (87.3%) 50 mg Etanercept (53.4%) Placebo group (7.3%)
NCT01474512	Psoriasis	Ixekizumab	Phase III	sPGA (12 wk) PASI 75 (12 wk)	sPGA responder rate: Ixekizumab 160 mg Q4W (76.4%) Ixekizumab 160 mg Q2W (81.8%) Placebo group (3.2%) PASI 75 rate: Ixekizumab 160 mg Q4W (82.6%) Ixekizumab 160 mg Q2W (89.1%) Placebo group (3.9%)
NCT01597245	Psoriasis	Ixekizumab	Phase III	sPGA (12 wk) PASI 75 (12 wk)	sPGA responder rate: Ixekizumab 80 mg Q4W (72.9%) Ixekizumab 80 mg Q2W (83.2%) 50 mg Etanercept (36.0%) Placebo group (2.4%) PASI 75 rate: Ixekizumab 80 mg Q4W (77.5%) Ixekizumab 80 mg Q2W (89.7%) 50 mg Etanercept (41.6%) Placebo group (2.4%)
NCT02529956	Psoriasis	Bimekizumab	Phase I	PASI 60	PASI 60 rate (over placebo): Bimekizumab 4/80 mg (> 80%) Bimekizumab 160 mg (> 85%) Bimekizumab 480/640 mg (> 94%)

ACR: American College of Rheumatology index; ASAS: Assessment of SpondyloArthritis International Society; PASI: Psoriasis Area and Severity Index; Q2W: every 2 weeks; Q4W: every 4 weeks; sPGA: Static Physician’s Global Assessment.

The clinical application of IL-17 inhibitors holds great promise, especially in the exploration of new indications. Currently, IL-17 inhibitors are primarily used in treating immune-related diseases such as psoriasis and AS (Yin et al., 2020; Thomas et al., 2024); however, they may also have significant therapeutic potential in other immune-mediated diseases. IL-17 is crucial in the pathogenesis of IBD, particularly in local immune responses in the gut. Therefore, further exploration of IL-17 inhibitors in these diseases may be worthwhile. Similarly, MS, an autoimmune disease of the CNS, is primarily driven by T-cell-mediated immune responses that lead to neuroinflammation. Research suggests that IL-17 may promote inflammation and myelin damage in MS. As a result, IL-17 inhibitors are being explored as a possible MS treatment.

With the development of precision medicine, the therapeutic effects of IL-17 inhibitors may be optimized through genetic screening and biomarker detection to enable more personalized treatment plans (Tan et al., 2024). Precision medicine aims to optimize treatment strategies by understanding the genetic background, immune system characteristics, and molecular mechanisms of diseases in patients. The activation of IL-17 signaling pathways may vary across different immune diseases, so genomics and biomarker testing could help predict patients’ responses to IL-17 inhibitors, enabling more personalized treatment approaches (Gu et al., 2009). For patients with poor responses or resistance, combination therapies involving IL-17 inhibitors and other immune-modulating drugs may provide new therapeutic opportunities. For example, combining IL-17 inhibitors with TNF-α inhibitors or IL-12/23 inhibitors may enhance efficacy by simultaneously targeting multiple immune pathways, especially in patients whose diseases cannot be effectively controlled by monotherapy (Costache et al., 2022). Furthermore, combining IL-17 inhibitors with small-molecule immune modulators (such as Janus kinase inhibitors) may also emerge as an effective strategy for treating immune-mediated diseases, warranting further investigation in future clinical studies (Costache et al., 2022).

To summarize, IL-17 inhibitors have broad application prospects not only in optimizing efficacy for existing indications but also in exploring new indications and combination treatment strategies in the future. Overall, IL-17 inhibitors have demonstrated notable effectiveness in treating various immune disorders, providing new hope for patients. However, treatment safety, resistance, and the realization of personalized therapies remain key areas for future research. With ongoing optimization of treatment strategies, the exploration of new indications, and long-term efficacy studies, IL-17 inhibitors are poised to play a growing role in the management of immune diseases.

## Conclusion and Perspectives

Over the past 25 years, IL-17 has transitioned from a relatively obscure cytokine family to a central player in immune regulation and a crucial therapeutic target for autoimmune diseases. This remarkable journey reflects a growing understanding of the critical function of IL-17 in modulating immune activity and driving the pathogenesis of a range of autoimmune conditions. Initially recognized for its participation in immune-related responses, the IL-17 family is crucial. The discovery of its potent effects on immune cell activation and tissue inflammation gave rise to the identification of Th17 cells as central contributors to autoimmune conditions like RA (Min et al., 2021; Schinocca et al., 2021; Yang et al., 2023b), psoriasis (Gupta et al., 2021; Fries et al., 2023), and MS (Zhao et al., 2021; Li et al., 2024a). Nevertheless, IL-17 cannot be solely synthesized by Th17 cells; other immune cell populations, including γδ T cells and ILCs, contribute to IL-17 cytokine production, which expands its impact on immune modulation and inflammatory processes.

In addition to IL-17A, the roles of other IL-17 family members in immune and inflammatory responses are increasingly elucidated. These cytokines not only function in conjunction with or in a complementary manner to IL-17A but may also exhibit independent physiological functions in different biological contexts. For example, IL-17B is believed to play a role in promoting embryogenesis. IL-17C is critical in epithelial cell immune responses, particularly in barrier tissues such as the gut, respiratory tract, and skin. IL-17D holds the potential for anti-infectious immunity, while IL-17E is crucial in defending against parasitic damage.

Grasping the cellular origins of IL-17 family members is essential for comprehending their involvement in the course of diseases. The predominant source of IL-17A and IL-17F is Th17 lineage cells, which give rise to naïve CD4^+^ T cells as a consequence of IL-6, TGF-β, and IL-23 (Harbour et al., 2020; Waśkiel-Burnat et al., 2021; Lin et al., 2022a). Nevertheless, other cell types, incorporating γδ T cells, can also lead to IL-17 production as a reaction to tissue inflammation. The indicated various cellular sources offer a complex network of immune regulation, and their interactions are crucial in diseases where IL-17 is an essential contributor to triggering inflammation. The discovery of these cellular contributors provides insight into the multifaceted nature of IL-17-driven diseases, emphasizing the need for precision in targeting IL-17 for therapeutic purposes.

One notable gap in the current literature concerns the IL-17D receptor. While much attention has been paid to IL-17A and IL-17F, IL-17D and its receptor remain less studied despite evidence suggesting their involvement in tissue-specific inflammatory responses. IL-17D appears to have distinct biological effects relative to other IL-17 family members, and its receptor represents a valuable therapeutic avenue for future research. Understanding the involvement of IL-17D across physiological and pathological contexts could open new avenues for targeted therapies, particularly in conditions where other IL-17 cytokines may not be as influential.

Cytokines from the IL-17 family, notably IL-17A, are implicated in the initiation of multiple autoimmune disorders. For instance, in RA, IL-17A promotes synovial inflammation and joint destruction by inducing the liberation of proinflammatory cytokines like TNF-α along with matrix MMPs that break down extracellular matrix components. Similarly, in psoriasis, IL-17A and its interactions with other immune pathways contribute to the hyperproliferation of keratinocytes and the formation of psoriatic plaques. In MS, Th17 cells with IL-17 production contribute to B damage to the BBB and the event triggering neuroinflammation, which drives disease progression. Although IL-17’s involvement in these diseases has been thoroughly investigated, there are still significant gaps in our understanding of its full range of functions, particularly in its interactions with other immune pathways, such as IL-23 and TNF signaling.

The efficacy of IL-17/IL-17R pathway modulation has been demonstrated in clinical trials, especially with monoclonal antibodies such as Secukinumab, Ixekizumab, and Brodalumab, which selectively inhibit IL-17A or its receptor. These therapies have demonstrated significant effectiveness in treating diseases like psoriasis, surpassing traditional non-inflammatory drugs and TNF inhibitors with respect to efficacy and safety. However, clinical trials have also uncovered significant side effects, including depression, increased risk of infections, and the exacerbation of autoimmune inflammation in some patients, highlighting the complexity of targeting IL-17 in therapeutic interventions. Autoimmunity remains a major concern, as the immunosuppressive properties of IL-17 inhibitors may trigger the emergence of additional autoimmune disorders, posing challenges to long-term treatment success.

Given these challenges, the next 25 years of IL-17 research are poised to explore new therapeutic opportunities. Advances in single-cell gene expression analysis, CRISPR technology, and other cutting-edge techniques will provide an in-depth understanding of the precise involvement of IL-17 in maintaining tissue balance, healing, and disease progression. These advancements may facilitate the discovery of novel biomarkers for more precise patient classification, enabling more personalized treatments. Furthermore, novel combination therapies targeting IL-17 in conjunction with other immune modulators may offer more effective and safer treatment strategies, improving outcomes for patients with autoimmune diseases.

Although this review systematically summarizes the biological functions of the IL-17 family in immune diseases and recent therapeutic advancements, several limitations remain. First, while the literature search covered multiple databases, it did not include preprints or non-English publications, potentially omitting emerging research and region-specific data. Second, studies on certain IL-17 family members (such as IL-17D and IL-17C) are still in the early stages, and limitations in elucidating their receptor mechanisms and pathological roles constrain the depth of discussion; thus, some conclusions require further experimental validation. Additionally, although the section on clinical translation focuses on monoclonal antibody-based therapies, discussions on small-molecule inhibitors, combination therapies, and resistance mechanisms remain insufficient, and a systematic analysis of long-term safety and patient heterogeneity is lacking. Finally, the pathological differences between certain animal models and human diseases have not been fully addressed, potentially affecting the reliability of mechanistic extrapolations. Future research should integrate multi-omics approaches and clinical big data to further elucidate the dynamic regulation of the IL-17 signaling network, thereby optimizing targeted strategies and advancing personalized therapies.

## Data Availability

*Not applicable*.
